# Microwave Spectra and Molecular Structures of the Gas-Phase Heterodimers Formed between Argon and 3,3,3-Trifluoropropene and between Acetylene and 3,3,3-Trifluoropropene

**DOI:** 10.1021/acs.jpca.6c00491

**Published:** 2026-03-24

**Authors:** Helen O. Leung, Mark D. Marshall, Pohakeaokahokuula G. Mawyer, Luke N. Kline

**Affiliations:** Department of Chemistry, Amherst College, P.O. Box 5000, Amherst, Massachusetts 01002-5000, United States

## Abstract

The gas-phase heterodimers formed by argon and acetylene with 3,3,3-trifluoropropene are investigated using *ab initio* calculations and Fourier transform microwave spectroscopy. Spectroscopic constants of the most abundant isotopologues of these two complexes, as well as their minor isotopologues containing a single ^13^C substitution, reveal their structures. Ar is located above the planar HCCCF cavity of the trifluoropropene so that it can interact with a maximum number of heavy atoms. Acetylene binds to the H atom of trifluoropropene geminal with the CF_3_ group, and lies in the symmetry plane of trifluoropropene. One of the acetylenic H atoms forms a bifurcated hydrogen bond with both out-of-plane fluorine atoms, and the acetylenic bond interacts with the geminal H atom. The proximity of this H atom to the CF_3_ group suggests that this motif is driven by electrostatic factors.

## Graphical abstract

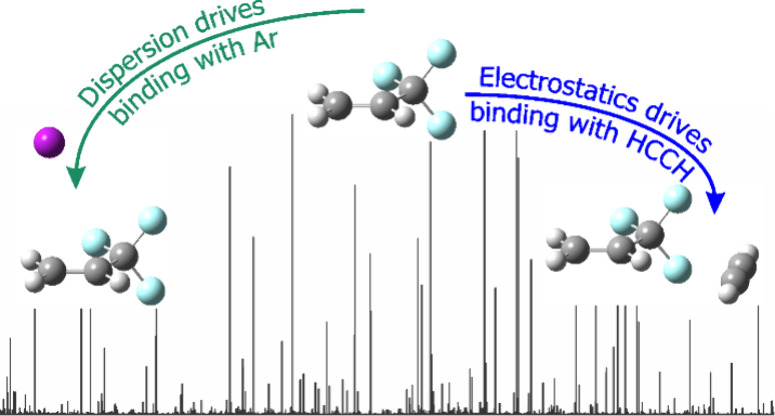

## I Introduction

Our series of studies on the intermolecular interactions between haloethylenes and simple protic acids have furnished abundant information. The haloethylenes we choose contain both nucleophilic and electrophilic atoms. Specifically, they contain F and/or Cl atoms and at least one H atom. (We use at most one Cl atom to avoid extensive splitting of spectral lines due to the complicated nuclear quadrupole hyperfine interaction that would arise from multiple Cl atoms in the haloethylenes.) The protic acids are hydrogen fluoride, hydrogen chloride, and acetylene, each of which also contains electrophilic and nucleophilic components. Interestingly, our work on 22 of these weakly bound complexes,^1^ combined with four from the Legon group,^2–6^ shows generally only three planar binding motifs. (One notable exception is the vinyl chloride-HCl complex, which is nonplanar and exhibits tunneling motion.)^7,8^ Collectively, the structures of these complexes reveal the manner in which electrostatic and steric factors in these systems are balanced.^1^

To expand our knowledge of how different functional groups participate either cooperatively or competitively in intermolecular interactions, we have extended our work to halopropene complexes. By replacing one of the atoms in the haloethylene subunit with a fluoromethyl (CH*_n_* F_3‑*n*_, *n* = 1, 2) group, we introduce additional nucleophilic F atoms which not only furnish extra sites for intermolecular interactions, but also inevitably change the electronic distribution of the entire molecule. Our goal is to observe how these modifications affect the interaction of the molecule with the same protic acid partners (HF, HCl, HCCH) and refine our understanding of the interplay between electrostatic and steric factors. We also use argon as a binding partner to find out how the halopropenes interact via dispersion interactions.

Thus, far, we have studied six halopropene monomers and their argon complexes. The configurations of these monomers are shown in Figure 1 with the three C atoms placed in the plane of the page, and the positions of the Ar atom above this plane are indicated for their argon complexes. Five of the halopropenes contain a CF_3_ group and a plane of symmetry formed by all atoms except two of the F atoms in the group. For four of the halopropenes, 2,3,3,3-tetrafluoropropene^9,10^ (Figure 1a), (*E*)-1,3,3,3-tetrafluoropropene^11^ (Figure 1b), (*Z*)-1,2,3,3,3-pentafluoropropene^12^ (Figure 1c), and (*E*)-1,2,3,3,3-pentafluoropropene^12^ (Figure 1d), the planar F atom in the CF_3_ group points toward the atom *cis* to the group, which is an H atom for the first three halopropenes. This orientation makes possible an intramolecular hydrogen bond between these two atoms, which is not possible for (*E*)-1,2,3,3,3-pentafluoropropene. The orientation of the CF_3_ group is different for (*Z*)-1-chloro-3,3,3-trifluoropropene^13^ (Figure 1e). Here, the out-of-plane F atoms in CF_3_ group point toward the chlorine atom so that the planar F atom can form an interaction with the H atom geminal to the CF_3_ group. The sixth species, 2,3,3-trifluoropropene, contains a CHF_2_ group and has two rotamers.^14^ In both rotamers, the fluoromethyl group orients in a similar fashion as the CF_3_ group in the fluoropropenes shown in Figure 1a–d, that is, with one of the atoms pointing toward the H atom *cis* to the CHF_2_ group. The lower energy rotamer, labeled as rotamer (i) (Figure 1f) does not have a plane of symmetry, although one of the F atoms in the CHF_2_ group is almost coplanar with the C atoms in the propene and forms an intramolecular hydrogen bond with an H atom connected to C1, the carbon atom with two C–H bonds. There is also another intramolecular hydrogen bond, albeit longer (and therefore, weaker), between the H atom in the CHF_2_ group and the F atom geminal to it. The higher energy rotamer of 2,3,3-trifluoropropene, labeled as rotamer (ii) (Figure 1g) has a plane of symmetry; the only out-of-plane atoms are the two F atoms in the fluoromethyl group. This rotamer has no intramolecular hydrogen bond.

**Figure 1:**
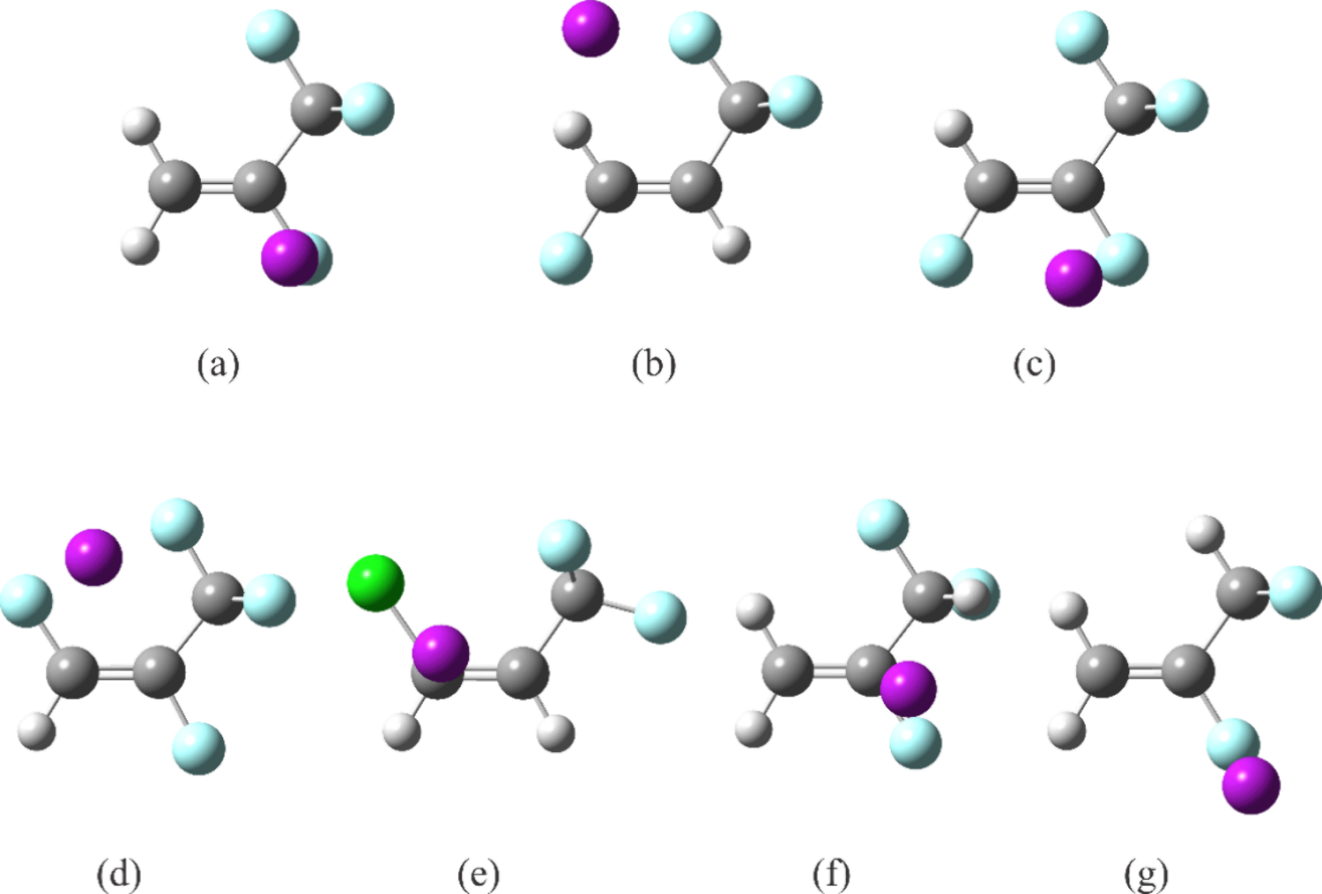
Experimental average structures for the argon complexes of (a) 2,3,3,3-tetrafluoropropene,^9,10^ (b) (*E*)-1,3,3,3-tetrafluoropropene,^11^ (c) (*Z*)-1,2,3,3,3-pentafluoropropene,^12^ (d) (*E*)-1,2,3,3,3-pentafluoropropene,^12^ (e) (*Z*)-1-chloro-3,3,3-trifluoropropene,^13^ (f) lower energy rotamer of 2,3,3-trifluoropropene, “rotamer (i)”,^14^ and (g) higher energy rotamer of 2,3,3-trifluoropropene, “rotamer (ii)”.^14^ Atom colors: C, dark gray; H, light gray; F: light blue, Cl: green, Ar: purple.

Turning to the argon complexes, Ar is positioned so that it can interact with the maximum number of heavy atoms in the halopropenes, as indicated in Figure 1. This is the same manner in which Ar is located to interact with their haloethylene counterparts, which are, if we replace each fluoromethyl group in Figure 1 [with the exception of Figure 1(b)] by an F atom, Ar-1,1-difluoroethylene,^15^ Ar-1,1,2-trifluoroethylene,^15^ and Ar-(*Z*)-1-chloro-2-fluoroethylene.^16^ Specifically, Ar is bound on one side of the ethylene plane above, respectively, the cavities formed by FCF, FCCF, and ClCCF. [Ar-(*E*)-1,2-difluoroethylene is the counterpart of (*E*)-1,3,3,3-tetrafluoropropene, part (b) of Figure 1. It should have a very small dipole moment; thus, its rotational spectrum has not been observed and the argon binding mode to this species has not yet been determined.] The observed binding motifs in these halopropenes and haloethylenes are of no surprise: argon is a structureless base and interacts via dispersion interactions, which are particularly effective when binding to heavy atoms.

The similarity observed in the argon complexes of halopropenes and their haloethylene counterparts is not realized in the acetylene complexes of (*Z*)-1-chloro-3,3,3-trifluoropropene and (*Z*)-1-chloro-2-fluoroethylene. There are two possible binding sites for HCCH in (*Z*)-1-chloro-2-fluoroethylene. Although the geminal F, H pair provides both a more nucleophilic halogen atom and a more electropositive H atom, HCCH prefers to bind to the Cl, H pair as a result of the relaxed steric requirement of Cl for a hydrogen bond as well as favorable electrostatic interaction between the acetylenic bond with the geminal H atom (Figure 2a), as explained in the following.^17^ The most negative electrostatic potential of Cl is on a band about the atom more or less perpendicular to the C–Cl bond whereas that of F is along the C–F bond and points away from it. Thus, the chlorine atom imposes a less stringent angular requirement for the hydrogen bond with HCCH (that is, a smaller CCl···H angle is possible without undue strain). Moreover, the Cl···H bond, which is weaker than a putative F···H bond, allows the hydrogen bond to bend more from linearity to enhance the interaction between the acetylenic bond and the H atom geminal to Cl. When the F atom in (*Z*)-1-chloro-2-fluoroethylene is replaced by a CF_3_ group, even though there are more electronegative F atoms with different steric requirements as well as the availability of the geminal Cl, H pair, HCCH adopts a novel binding mode with the acetylenic bond interacting with the two H atoms in the halopropene (Figure 2b). This configuration arises likely because the presence of four halogen atoms can weaken the nucleophilicity of each while potentially strengthening the electropositivity of the hydrogen atoms. Indeed, a restrained electrostatic potential analysis^18^ obtained from Multiwfn^19^ using the 6–311++G­(2d,2p) basis set utilized for this study (see below) indicates that while the partial positive charges on the hydrogen atoms geminal to the chlorine atom in (*Z*)-1-chloro-3,3,3-trifluoropropene and (*Z*)-1-chloro-2-fluoroethylene are similar, the partial positive charge on the hydrogen atom geminal to the fluorine atom or trifluoromethyl group increases from +0.13 to +0.19 in going from (*Z*)-1-chloro-2-fluoroethylene to (*Z*)-1-chloro-3,3,3-trifluoropropene. The halopropene therefore becomes a stronger acid than HCCH and donates its H atoms to the electron rich triple bond.

**Figure 2:**
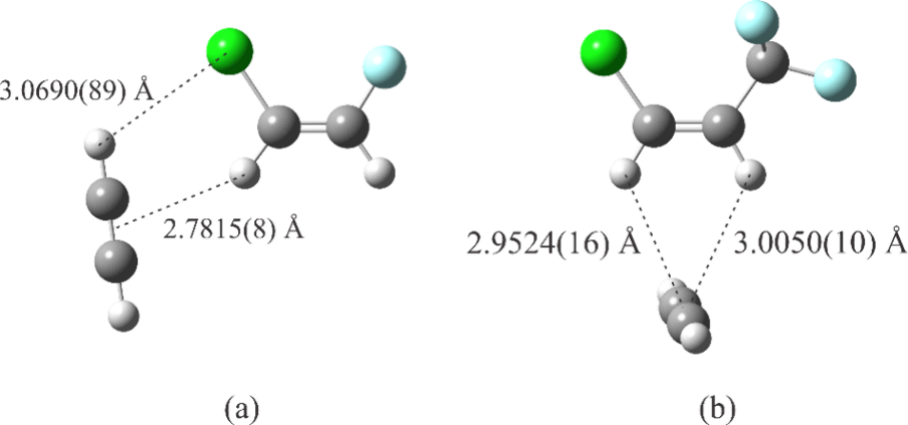
Experimental average structures for the HCCH complexes of (a) (*Z*)-1-chloro-2-fluoroethylene^17^ and (b) (*Z*)-1-chloro-3,3,3-trifluoropropene.^41^ Atom colors: C, dark gray; H, light gray; F: light blue, Cl: green.

Because there are other halogen atoms present in the halopropene systems thus far, it is difficult to tease out the role played solely by a fluoromethyl group in intermolecular interactions and by extension, the effects exerted by the other functional groups. Thus, we turn to 3,3,3-trifluoropropene (TFP), which can be thought of as deriving from the replacement of the F atom in vinyl fluoride with a CF_3_ group. The rotational spectrum of the ground vibrational state of TFP—the state of interest in our work—has been previously studied,^20–22^ with an extensive microwave region (8–75 GHz) covered for the most abundant isotopologue. The experimental structure (*r*_0_) of the molecule derived using the mixed estimation technique by Alonso et al.^22^ shows a configuration with a symmetry plane that contains one of the F atoms of the CF_3_ group (Figure 3). This atom forms an intramolecular hydrogen bond (2.429 Å) with the H atom connected to C1 while the other two F atoms form a bifurcated hydrogen bond (2.682 Å) with the H atom connected to C2. We report here our study on two of its complexes, Ar-TFP and HCCH-TFP, and compare their structures with their vinyl fluoride counterparts and other analogous halopropene complexes. In the course of our work, we have also extended the rotational spectrum of the most abundant isotopologue of TFP down to 2 GHz and augmented the number of transitions for the singly substituted ^13^C isotopologues from six as reported in Alonso et al.^22^ to a total of 106 reported here.

**Figure 3:**
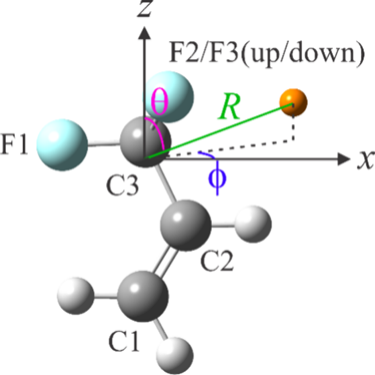
Atom labeling scheme for 3,3,3-trifluoropropene (TFP) and the coordinate system (with the origin at the center of mass of the halopropene) used to construct the interaction potential surfaces for its argon and acetylene complexes. The orange sphere represents Ar for the former surface and the center of mass of HCCH for the latter one. It forms a polar angle (θ) with the *z* axis and an azimuthal angle with the *x* axis. Atom colors: C, dark gray; H, light gray; F: light blue.

## II *Ab Initio* Calculations

To inform our search for the rotational spectra of Ar-TFP and HCCH-TFP, we construct an interaction potential surface between the subunits of each complex and explore the configurations of the species at each local minimum. The geometry of TFP is fixed to that determined by Alonso et al.^22^ and we use its inertial axis system as a reference, labeling the *a*, *b*, *c* axes as *z*, *x*, *y* axes (in accordance with the *I* ^*r*^ convention), respectively (Figure 3). For the Ar complex, we scan the position of the Ar atom at a polar angle (θ) from 5° to 175° and a dihedral angle (ϕ) from −180° to 180°, each in 10° increments while optimizing its distance (*R*) from the origin. For the HCCH complex, we fix HCCH at its average ground-state structure,^23^ scan the position of its center of mass and optimize its distance from the origin in the same manner as we do for the Ar complex, but this time, we also optimize the orientation of HCCH. Although density functional theory can perform quite well for these systems and is less computationally expensive, we employ the MP2/6–311++G­(2d,2p) level using GAUSSIAN 16 ^24^ as we have done in earlier studies for haloethylene and halopropene complexes, for two reasons. First, the use of the same level of theory allows for easy comparisons among analogous molecular systems. Second, this level of theory typically provides better relative energies for the isomers of a complex as well as satisfactory estimates of the values of the rotational constants for the species we have observed.

The interaction potential energy contour diagrams are shown in Figure 4. For the Ar complex, there are two pairs of equivalent minima and one unique minimum labeled in order of increasing potential energy as (a)–(c), and for the HCCH complex, two unique minima and one equivalent pair labeled as (i)–(iii), also in order of increasing energy. The equivalent pairs are a consequence of the symmetry at ϕ = 0° arising from the symmetry plane of TFP. The scales of the two surfaces are quite different, with the surface for the Ar complex being much shallower.

**Figure 4:**
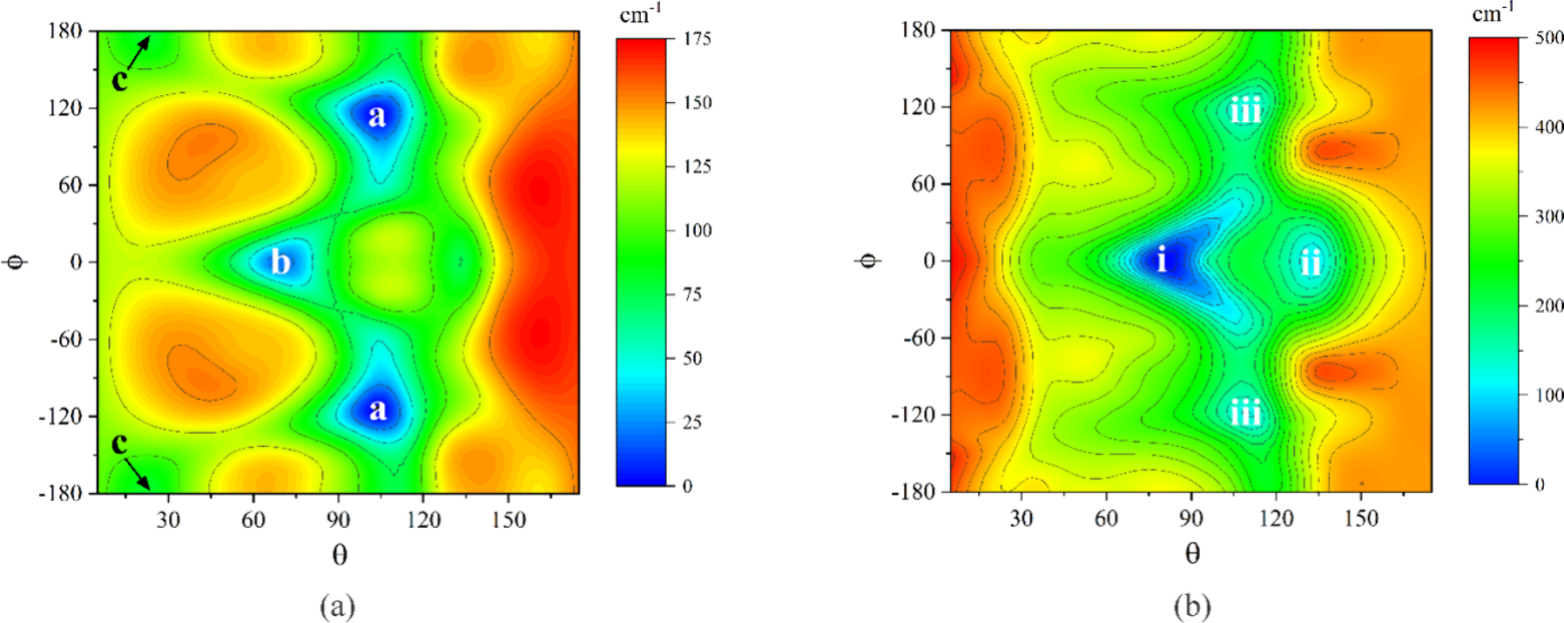
Interaction potential energy contour diagrams formed by 3,3,3-trifluoropropene with (a) Ar and (b) HCCH.

The optimized structures and their relative energies corresponding to the potential minima for Ar-TFP are shown in Figure 5. For each of the Ar-TFP isomers, Ar is positioned to interact with multiple heavy atoms. The atomic positions of each isomer in its principal axis system, with and without the counterpoise correction for basis set superposition error (BSSE),^25^ the distances between Ar and the heavy atoms in the three isomers, and the rotational constants and dipole moment components for the isomers are available as Supporting Information.

**Figure 5:**
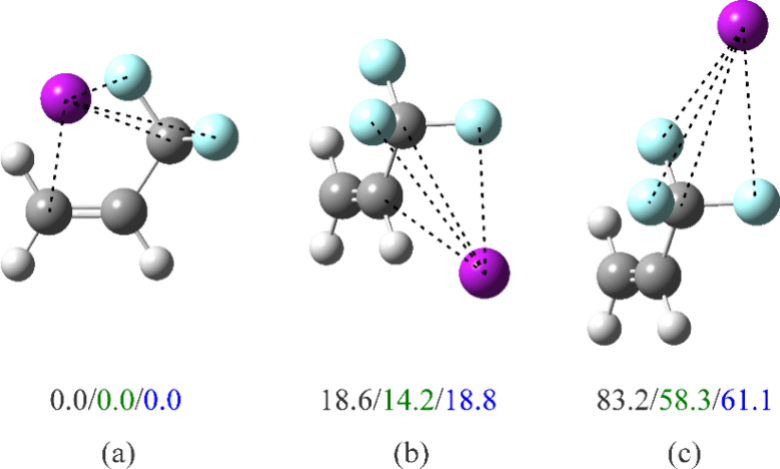
Optimized structures and relative energies (in cm^–1^) of three isomers of Ar-TFP corresponding to the minima in the potential energy contour plot in Figure 4a. Energies in black include neither the basis set superposition error (BSSE) nor zero-point energy corrections, those in green include only the BSSE correction, and those in blue, both BSSE and zero-point corrections. Those interaction lengths that are no more than ∼10% longer than the van der Waals contact^42^ are indicated by dashed lines. Atom colors: C, dark gray; H, light gray; F: light blue; Ar: purple.

When the structures corresponding to the minima in the potential energy contour plot for HCCH-TFP are optimized, they show three isomers (Figure 6), with Isomer (i) lower in energy by ∼130 cm^–1^ than Isomers (ii) and (iii), which are similar in energy. Isomer (i) represents a novel structure in which HCCH lies in the symmetry plane of TFP and forms two sets of interactions: (1) a bifurcated hydrogen bond with the two out-of-plane F atoms in TFP and (2) the electron rich acetylenic bond with the H atom geminal to the CF_3_ group. The configuration of Isomer (ii) is similar to that observed for HCCH-1-chloro-3,3,3-trifluoropropene where the HCCH subunit is perpendicular to the symmetry plane of TFP and interacts with two H atoms of TFP via the acetylenic bond. Structure (iii) corresponds to one of the two equivalent minima in the potential energy contour plot: HCCH lies on one or the other side of the symmetry plane of TFP. Here, HCCH forms hydrogen bonds with two F atoms, one in-plane, the other out-of-plane, in the CF_3_ group. The interaction is shorter (hence stronger) with the out-of-plane F atom than the F atom in the plane. The acetylenic bond is positioned somewhat far away from the H atom *cis* to the CF_3_ (3.583 Å) and from the ethylenic bond (4.089 Å), hence leading to weaker interactions. The rotational constants and dipole moment components of each isomer and the atomic positions of each isomer in its principal axis system are available as Supporting Information.

**Figure 6:**
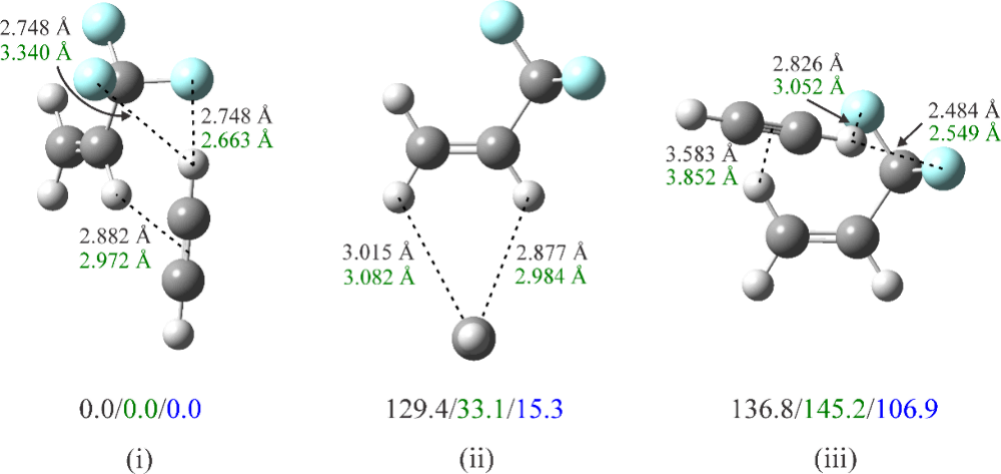
Optimized structures and relative energies (in cm^–1^) of three isomers of HCCH-TFP corresponding to the minima in the potential energy contour plot in Figure 4b. Energies in black include neither the basis set superposition error (BSSE) nor zero-point energy corrections, those in green include only the BSSE correction, and those in blue, both BSSE and zero-point corrections. Equilibrium intermolecular distances without correcting for BSSE are indicated in black, and those including the correction are indicated in green. Atom colors: C, dark gray; H, light gray; F: light blue.

The rotational constants and dipole moment components for the three isomers are all very different, and we can easily distinguish among them if they are observed. Isomer (i) is predicted to be the lowest in energy, but the energies of Isomers (ii) and (iii) are not so high that they can be excluded with confidence when embarking upon the experimental search for the rotational spectrum.

## III Experiment

We use a broadband, chirped-pulse Fourier transform microwave spectrometer operating from 2.0 to 18.1 GHz to observe the spectra of the four isotopologues of Ar-TFP (the most abundant species and three isotopologues singly substituted with ^13^C in natural abundance) as well as the most abundant isotopologue of HCCH-TFP. Although certainly present in the sample, the signals for the HCCH-TFP isotopologues singly substituted with ^13^C in natural abundance are, however, too weak to be assigned in the broadband spectrum; we thus turn to the more sensitive narrowband Balle-Flygare spectrometer to search for and record them. A sample of 2/3–1% of TFP in Ar was used for the monomer and Ar complex, while a mixture of 2/3–1% of each TFP and HCCH in Ar was used for the HCCH complex, both in the broadband and narrowband instruments. The chirped pulse spectrometer uses two pulsed valves, while the Balle-Flygare spectrometer uses one, each with a 0.8 mm diameter nozzle. The backing pressure is 2–3 atm.

The broadband spectrum is collected in four segments: 2.0–6.0, 5.6–10.1, 10.1–14.1, and 14.1–18.1 GHz. The first segment utilizes a 1.0–3.0 GHz chirped microwave pulse of 1 μs duration that is actively doubled and then amplified to 500–700 W of power with a traveling wave tube amplifier to polarize the sample. For the other three bands, the polarization pulse is obtained by mixing a chirped microwave pulse of 4 μs duration and the appropriate frequency range with the output of phase-locked dielectric resonator oscillators at 10.6, 14.6, and 18.6 GHz, respectively. After isolation of the lower sideband, the pulse is amplified to 20–25 W of power with a solid-state amplifier. After polarization, the subsequent free-induction decay (FID) is digitized at 50 Gs s^–1^ starting 0.5 μs after the end of the excitation pulse and continuing for 20 μs. Ten polarization-digitization cycles are performed for each 800 μs opening of the pulsed valves, which operate at 4 Hz, and approximately 1,455,000 to 1,800,000 FIDs are averaged for each segment. The averaged FID is apodized, zero-filled and Fourier transformed, as described previously,^16^ to give a frequency domain spectrum with a resolution element of 11.92 kHz and typical line widths of 125 kHz. The spectra from the four segments are then stitched together. We estimate the frequency measurement uncertainty to be 5–10 kHz using this procedure. The quality of our broadband spectrum taken with a sample of TFP and HCCH in Ar is illustrated in Figure 7 in which transitions due to the most abundant isotopologues of TFP and HCCH-TFP and four isotopologues of Ar-TFP can be observed.

**Figure 7:**
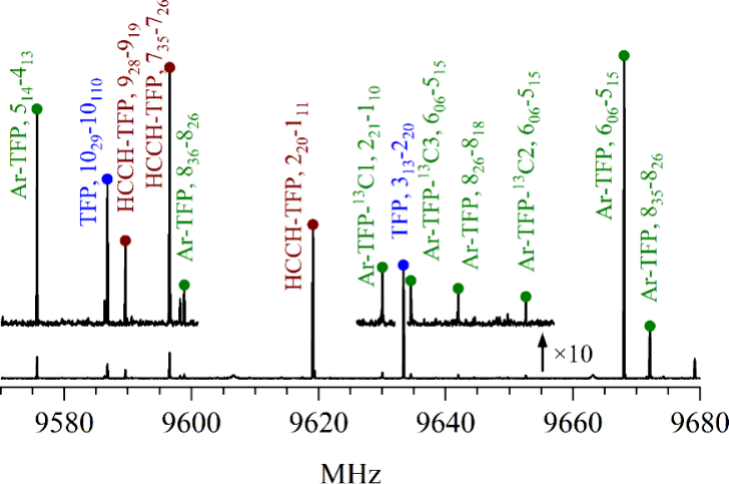
110 MHz portion of the broadband spectrum obtained using a mixture of TFP and HCCH in Ar. The upper trace has a 10-fold magnification to better illustrate the weaker lines. The assigned transitions are marked and labeled. The transitions due to the Ar-containing species are typically more intense in the spectrum taken using only TFP in Ar (that is, without HCCH in the mixture).

The narrowband spectrometer^16,26^ operates in the 5–21 GHz region. The time domain signal is background-corrected and digitized for 1024 data points and zero-filled to a 2048-point record length before Fourier transformation, resulting in a frequency domain spectrum with a 4.8 kHz resolution. Because the nozzle is mounted behind one of the mirrors that forms the Fabry-Pérot cavity, the molecular beam is parallel to the mirror axis. As a result, each transition appears as a Doppler doublet. The rest frequency of the transition is the mean frequency of the Doppler components.

## IV Results

### Spectral Analysis

The broadband spectra are assigned using Kisiel’s AABS program.^27^ The transitions for the isotopologues of the TFP monomer, Ar-TFP, and HCCH-TFP are analyzed using the Watson *S*-reduced Hamiltonian in the *I* ^*r*^ representation^28^ and Pickett’s nonlinear least-squares SPFIT program.^29^ In the case of the TFP isotopologues, the Watson *A*-reduced Hamiltonian in the *I* ^*r*^ representation^28^ was also employed to better compare the spectroscopic constants with those reported by Alonso et al.^22^ Tables of observed and calculated transition frequencies with assignments for the all species are in Supporting Information. The rms deviation for each fit when the transitions are obtained from the broadband spectrometer is 3.7–6.3 kHz and is 1.0–1.6 kHz from the narrowband spectrometer. These values are commensurate with the resolution element of each instrument.

#### 3,3,3-Trifluoropropene

We measure rotational transitions, both *a*- and *b*-type, due to four isotopologues of the TFP monomer in the ground vibrational state. For the most abundant species, Alonso et al.^22^ observe a greater number of transitions across a much broader range of frequencies (albeit not extending to the 2–8 GHz region) and quantum numbers. Regardless, their rotational constants and those determined by us (using the *A* reduced Hamiltonian) agree excellently, differing by no more than 1.7–4.4 kHz. For the isotopologues singly substituted with ^13^C, we observe a greater number of transitions than Alonso et al.^22^ and also *b*-type transitions not observed in that earlier work. We can therefore establish the *A* rotational constants more precisely than previously as well as determine four of the five quartic centrifugal distortion constants. Nevertheless, the *B* and *C* constants determined here and earlier differ by no more than 3 kHz. Of particular importance to our work, the high quality of the structure of TFP determined by Alonso et al. is demonstrated in that it reproduces the more precise *A* rotational constants of all four isotopologues determined here even better than it does those reported earlier where the precision was limited by the absence of *b*-type transitions. The structure also reproduces the *B* and *C* constants determined by both works to 0.01–0.23 MHz.

#### Ar-3,3,3-Trifluoropropene

All three types of transitions are observed for the most abundant isotopologue of Ar-TFP. In general, *b*-type transitions are most intense, and in fact, for the isotopologues singly substituted with ^13^C, are the majority of those observed. For all species, we determine the rotational constants, all 5 quartic centrifugal distortion constants, and for the most abundant isotopologue, because we are able to access a relatively large *J* range of 0–18 and *K*_*a*_ range of 0–5, we can also determine 3 sextic centrifugal distortion constants (Table 1). The rotational constants of the most abundant species and intensities of the transitions agree best with the theoretical values for Isomer (a).

**Table 1:** Spectroscopic Constants (in MHz, Unless Otherwise Noted) for Four Isotopologues of Ar-TFP^a^,^b^

	Ar–CH_2_CHCF_3_	Ar–^13^CH_2_CHCF_3_	Ar–CH_2_^13^CHCF_3_	Ar–CH_2_CH^13^CF_3_
*A*	3003.64088(17)	2923.75565(71)	2973.57810(78)	3003.13186(62)
*B*	991.865340(69)	991.59586(37)	988.94420(36)	989.02061(33)
*C*	866.022410(68)	858.96930(26)	862.32169(25)	863.79575(30)
*D*_*J*_/10^–3^	2.48278(59)	2.4383(37)	2.4646(36)	2.4703(34)
*D*_*JK*_/10^–3^	13.0958(29)	12.966(14)	13.133(18)	13.026(12)
*D*_*K*_/10^–3^	–9.7445(68)	–9.555(65)	–10.021(64)	–9.588(58)
*d*_1_/10^–3^	–0.37245(20)	–0.3896(20)	–0.3708(19)	–0.3719(16)
*d*_2_/10^–3^	–0.040501(15)	–0.04502(66)	–0.04142(93)	–0.03989(53)
*H*_*J*_/10^–6^	–0.0494(16)	[−0.0494]	[−0.0494]	[−0.0494]
*H*_*JK*_/10^–6^	–0.443(12)	[−0.443]	[−0.443]	[−0.443]
*h*_1_ ^c^/10^–6^	–0.01017(60)	[−0.01017]	[−0.01017]	[−0.01017]
no. of rot. transitions	320	51	40	50
no. of *a* type	59	0	0	0
no. of *b* type	177	51	40	49
no. of *c* type	84	0	0	1
*J* range	0–18	0–9	0–9	0–8
*K*_*a*_ range	0–5	0–3	0–3	0–3
rms/kHz	3.66	5.38	4.93	5.01

a 1σ standard deviations in the parameters are given in parentheses.

b The value of an undeterminable centrifugal distortion constant for each of the isotopologues singly substituted with ^13^C is fixed to that for the most abundant isotopologue and enclosed by square brackets.

c The inclusion of *h*_1_ for the most abundant species decreases the rms from 5.04 kHz to 3.66 kHz. Nevertheless, this gives a correlation of −0.976 with *d*_1._ A comparison of the spectroscopic constants obtained with and without the inclusion of *h*_1_ is included in the Supporting Information.

#### HCCH-3,3,3-Trifluoropropene

Only *a*- and *b*-type transitions are observed for the most abundant isotopologue of HCCH-TFP. The experimental rotational constants (Table 2) agree best with the theoretical values for Isomer (i). The agreement also extends to the relative intensity of the transitions: the observed *b*-type transitions are in general stronger, in accordance with a much greater dipole moment component along the *b* axis than that along the *a* axis in Isomer (i). [The values of the *c* component are 0 and 0.019 D (a vanishingly small value), respectively, without and with the inclusion of BSSE correction.] Once again, relatively large *J* (0–15) and *K*_*a*_ (0–5) ranges are observed, but for this complex, only 5 quartic centrifugal distortion constants are necessary to fit the spectroscopic data, indicating that this species likely exhibits less large amplitude motion than the argon complex. Only *b*-type transitions for the 5 isotopologues singly substituted with ^13^C are observed, and the spectroscopic constants are listed in Tables 2 and 3.

**Table 2:** Spectroscopic Constants (in MHz, Unless Otherwise Noted) for the Most Abundant HCCH-TFP and Two Isotopologues Singly Substituted with ^13^C in the HCCH Subunit^a^

	CH_2_CHCF_3_–HCCH	CH_2_CHCF_3_–H^13^CCH	CH_2_CHCF_3_–HC^13^CH
*A*	2841.57917(34)	2838.99375(26)	2836.77887(46)
*B*	1082.41236(14)	1062.53613(14)	1052.28676(25)
*C*	909.94424(13)	895.641856(73)	888.10202(13)
*D*_*J*_/10^–3^	1.34736(90)	1.3016(12)	1.3129(18)
*D*_*JK*_/10^–3^	10.4877(36)	10.366(11)	9.827(17)
*D*_*K*_/10^–3^	–9.881(10)	–9.574(24)	–9.177(45)
*d*_1_/10^–3^	–0.25135(19)	–0.2402(71)	–0.2417(12)
*d*_2_ ^b^/10^–3^	–0.022073(75)	[−0.022073]	[−0.022073]
no. of rot. transitions	151	24	22
no. of *a* type	20	0	0
no. of *b* type	131	24	22
*J* range	0–15	1–9	1–9
*K*_*a*_ range	0–5	0–3	0–3
rms/kHz	6.27	0.98	1.50

a 1σ standard deviations in the parameters are given in parentheses.

b The value of *d*_2_ for each of the isotopologues singly substituted with ^13^C is fixed to that for the most abundant isotopologue and enclosed by square brackets.

**Table 3:** Spectroscopic Constants (in MHz, Unless Otherwise Noted) for the HCCH-TFP Isotopologues Singly Substituted with ^13^C in the Propene Subunit^a^

	^13^CH_2_CHCF_3_–HCCH	CH_2_^13^CHCF_3_–HCCH	CH_2_CH^13^CF_3_–HCCH
*A*	2771.37072(48)	2820.69899(42)	2840.46406(35)
*B*	1078.40647(29)	1082.02214(24)	1080.87568(20)
*C*	899.78378(13)	907.51733(11)	908.727200(90)
*D*_*J*_/10^–3^	1.2969(23)	1.3324(19)	1.3435(15)
*D*_*JK*_/10^–3^	10.103(18)	10.369(16)	10.426(13)
*D*_*K*_/10^–3^	–9.219(48)	–9.657(41)	–9.753(34)
*d*_1_/10^–3^	–0.2471(14)	–0.2506(12)	–0.25020(95)
*d*_2_ ^b^/10^–3^	[−0.022073]	[−0.022073]	[−0.022073]
no. of rot. transitions	23	22	23
no. of *a* type	0	0	0
no. of *b* type	23	22	23
*J* range	1–9	1–9	1–9
*K*_*a*_ range	0–3	0–3	0–3
rms/kHz	1.59	1.38	1.15

a 1σ standard deviations in the parameters are given in parentheses.

b The value of *d*_2_ for each of the isotopologues singly substituted with ^13^C is fixed to that of the most abundant isotopologue and enclosed by square brackets.

### Structure Determination

#### Ar-3,3,3-Trifluoropropene

The experimental rotational constants, *A*, *B*, and *C*, for the most abundant isotopologue of Ar-TFP agree very well with those for the theoretical equilibrium structure of Isomer (a), with the respective theoretical values differing from the experimental values (experimental – theoretical) by 17.6, −18.1, −15.0 MHz (0.6%, −1.8%, −1.7%) when BSSE correction is not considered. When the correction is applied, the differences are −9.4, 65.9, and 48.0 MHz (−0.3%, 6.6%, and 5.4%). In our experience, there is no consistency regarding whether the theoretical rotational constants derived with or without BSSE correction agree better with experimental values. This is not surprising, because both sets correspond to an equilibrium structure while our experimental constants reflect an average structure. We have found, however, that both zero-point and BSSE corrections are generally necessary to correctly identify the lowest-energy isomer. Although not the case for Ar-TFP and HCCH-TFP, this is particularly important when the energy ordering of the isomers changes before and after the corrections.

Fixing the structure of TFP to that determined by Alonso et al.,^22^ we need only three geometric parameters to locate the argon atom. We fit the length of Ar–F1, the angle Ar–F1–C3, and the dihedral angle Ar–F1–C3–C2, to the three moments of inertia of each of the four isotopologues using Kisiel’s strfit program.^30^ The rms deviation is 0.088 u Å.^2^ The resulting structure is shown in Figure 8a, the principal coordinates of Ar and the C atoms in Table 4, and using Kisiel’s EVAL program,^31,32^ the distances between Ar and the heavy atoms are calculated and made available in the Supporting Information along with the principal coordinates of all atoms.

**Table 4:** Coordinates of the Carbon and Argon Atoms in Ar-TFP Determined from a Structure Fit and From Kraitchman Analysis^a^

	*a*/Å	*b*/Å	*c*/Å
from structure fit			
C1	–0.4415(19)	2.15877(45)	–0.02529(9)
C2	–1.1658(53)	1.1999(15)	0.5447(37)
C3	–1.22308(27)	–0.19498(26)	0.02414(21)
Ar	2.68827(14)	–0.15633(43)	0.01336(9)
substitution coordinates^b^			
C1	–0.4061(37)	2.15588(70)	unphysical
C2	–1.0739(14)	1.1635(13)	0.5997(25)
C3	–1.2151(12)	–0.1842(82)	unphysical
Ar	2.68849(56)	–0.1558(96)	0.01(11)

a 1σ standard deviations (for the structure fit) and Costain errors^40^ (for the substitution coordinates) in the parameters are given in parentheses.

b Although only the absolute values of the substitution coordinates can be determined from the Kraitchman analysis, the relative signs for most coordinates are assigned using physically reasonable atomic distances. The only exception is the substitution *c* coordinate of Ar; it is ill-determined, and thus the relative sign is not relevant.

**Figure 8:**
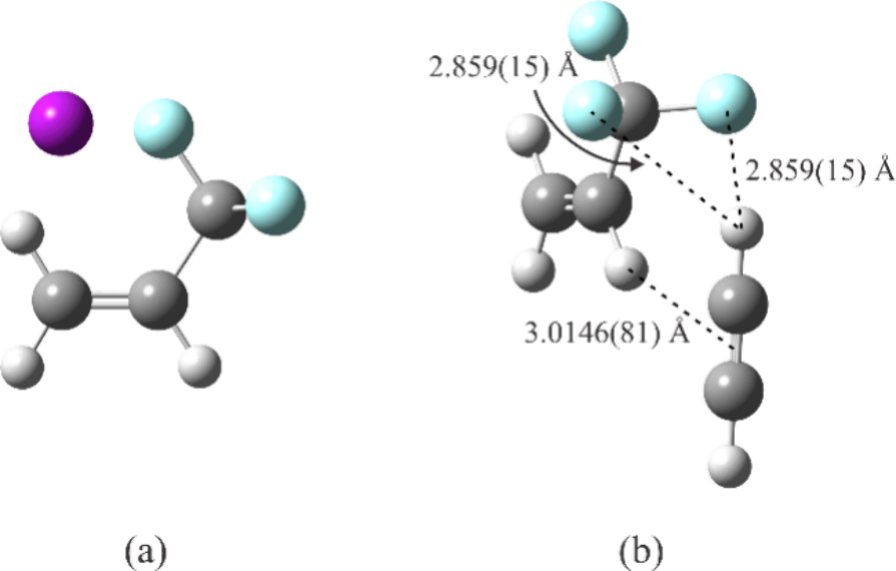
Experimental structures of (a) Ar-TFP and (b) HCCH-TFP.

We also use a Kraitchman analysis^33^ to determine the positions of the C and Ar atoms in the principal axis system of the most abundant isotopologue. The coordinates thus obtained are a good approximation to their equilibrium coordinates. Using the most abundant isotopologue as the parent, the absolute values of the coordinates for each C atom can be calculated by comparing the moments of inertia of the isotopologue singly substituted with ^13^C in that position and those of the most abundant species. The position of Ar can also be determined by considering the most abundant TFP monomer as containing a single substitution of a “mass 0” Ar.^34,35^ The substitution coordinates thus obtained are listed in Table 4. The unphysical values of the *c* coordinates of C1 and C3 indicate that these atoms locate very close to the *a-b* plane, which is consistent with the average structure of the complex. The average and substitution coordinates agree well, differing by no more than 0.09 Å. It is remarkable that the difference is no more than 0.003 Å for the Ar coordinates, even for the poorly determined *c* substitution coordinate.

#### HCCH-TFP

Once again, theory is helpful to us in identifying and assigning the transitions due to the most abundant isotopologue of HCCH-TFP. The experimental rotational constants for the most abundant isotopologue show the best agreement with those predicted for Isomer (i). However, this time, except for the value of *A*, the agreement is better for the theoretical values that include the BSSE correction than those without it. The differences (experimental – theoretical) in the values of *A*, *B*, *C* are 20.6, 24.4, 14.9 MHz (0.7%, 2.3%, 1.6%) with BSSE correction and are −0.4, −49.6, −34.1 (−0.01%, −4.6%, −3.7%) without BSSE correction.

Before we determine the structure of the complex, it is useful to find out if the HCCH subunit is in the symmetry plane of TFP or not, as suggested by the equilibrium structure without and with BSSE correction, respectively. The second moment *P*_*cc*_ $\left[P_{cc}=\underset{i}{\sum }m_{i}c_{i}^{2}=\left(I_{a}+I_{b}-I_{c}\right)/2\right]$ is ideal for this purpose. If HCCH is in the symmetry plane, then the only out-of-plane atoms are the two F atoms in the TFP subunit. As such, the values of *P*_*cc*_ for all the isotopologues of HCCH-TFP should be the same, and furthermore, they should be the same as those for the isotopologues of the TFP monomer. These are indeed the case. The values of *P*_*cc*_ for the six isotopologues of HCCH-TFP are between 44.662 and 44.681 u Å^2^ and those for the four isotopologues of TFP are between 44.321 and 44.328 u Å.^2^ The similarity between the values among the isotopologues of each species suggests that they contain the same out-of-plane atoms. The slightly greater difference between the two sets of species indicates that the TFP monomer and the TFP-HCCH complex exhibit marginally different zero-point motions.

Having established that HCCH is in the symmetry plane of TFP, and fixing the two subunits to their average structures,^22,23^ we need only three parameters to determine the structure of the complex: a distance between the two subunits and the angular orientation of each. Instead of using the moments of inertia of the isotopologues to determine the structure of the complex, we choose to use the second moments because, as shown earlier, one of them, namely, *P*_*cc*_, is useful in characterizing the position of HCCH relative to the symmetry plane of TFP. We use Kisiel’s strfit program^30^ to fit the structural parameters to the other two second moments, *P*_*aa*_ $\left[P_{aa}=\underset{i}{\sum }m_{i}a_{i}^{2}=\left(I_{b}+I_{c}-I_{a}\right)/2\right]$ and *P*_*bb*_ $\left[P_{bb}=\underset{i}{\sum }m_{i}b_{i}^{2}=\left(I_{a}+I_{c}-I_{b}\right)/2\right]$ for the six isotopologues to arrive at the experimental structure of the complex (Figure 8b). The rms deviation for the fit is 0.067 uÅ.^2^ The relevant bond distances indicated in Figure 8b are calculated using Kisiel’s EVAL program.^31,32^ (The principal coordinates of all atoms are available in Supporting Information.)

Once again, we use the most abundant isotopologue as the parent to carry out a Kraitchman analysis to determine the coordinates of the C atoms, which are given in Table 5. The *c* substitution coordinates for the C atoms in the TFP subunit are unphysical, consistent with the fact that they lie in the *a*-*b* plane. Those for the C atoms in the HCCH subunit are small and not well determined, which is consistent with the earlier argument that they lie in the symmetry plane of the TFP subunit. The *a* and *b* coordinates for all C atoms, determined using the structure fit and via a Kraitchman analysis, agrees quite well, differing by 0.002–0.066 Å.

**Table 5:** Coordinates of the Carbon Atoms in HCCH-3,3,3-Trifluoropropene Determined from a Structure Fit and from Kraitchman Analysis^a^

	*a*/Å	*b*/Å	*c*/Å
from structure fit^b^			
C1	–1.382(12)	–2.1231(39)	0
C2	–0.4643(68)	–1.1605(14)	0
C3	–0.8063(16)	0.28977(60)	0
C4	2.9612(58)	0.39577(64)	0
C5	3.6826(60)	–0.5736(85)	0
substitution coordinates^c^			
C1	–1.3158(11)	–2.13815(70)	unphysical
C2	–0.4106(37)	–1.1506(13)	unphysical
C3	–0.8197(18)	0.2747(55)	unphysical
C4	2.95940(51)	0.3920(28)	0.118(13)
C5	3.66245(41)	–0.5587(27)	0.065(23)

a 1σ standard deviations (for the structure fit) and Costain errors^40^ (for the substitution coordinates) in the parameters are given in parentheses.

b C4 and C5 are the C atoms in the HCCH subunit, which is fixed to be in the same plane as the symmetry plane of TFP. C4 is the C atom connected to that H atom that forms a bifurcated hydrogen bond with the two F atoms.

c Although only the absolute values of the substitution coordinates can be determined from the Kraitchman analysis, the relative signs for most coordinates are assigned using physically reasonable atomic distances. The only exceptions are the substitution *c* coordinates of C4 and C5; they are small and not well determined, and thus the relative signs are not relevant.

## V Discussion

Using argon as a carrier gas, the many-body collisions that form weakly bound complexes typically relax them to their lowest energy arrangement;^36–38^ thus, the observed rotational spectra in this work for Ar-TFP and HCCH-TFP represent their respective lowest energy isomers. It is noteworthy that even though several isomers for each complex are predicted to be so close in energy, theory was able to identify the most stable isomer in the present cases.

To look at how TFP interacts with Ar and HCCH, and more broadly, to compare these interactions with those of other halopropenes, it is instructive to map the electrostatic potential of each halopropene onto its total electron density surface. These surfaces are calculated at the MP2/6–311++G­(2d,2p) level and shown in Figure 9 at the same value of electron density. In the absence of other substituents in the ethylenic moiety, it is not surprising that the F atoms in TFP are very negative. They do, however, become less negative when additional F substituents are present. For the two rotamers of 2,3,3-trifluoropropene, the F atoms in the fluoromethyl group and that geminal to the group are all quite negative. In all these fluoropropenes with a CF_3_ or a CHF_2_ group, if a planar (or almost planar) F atom of the fluoromethyl group exists, it is less negative than the other F atoms in the group. This is likely because each out-of-plane C–F bond is part of the π system, and therefore, can withdraw electrons more effectively from the rest of the molecule through hyperconjugation. The in-plane F atom, on the other hand, is not part of the π system.

**Figure 9:**
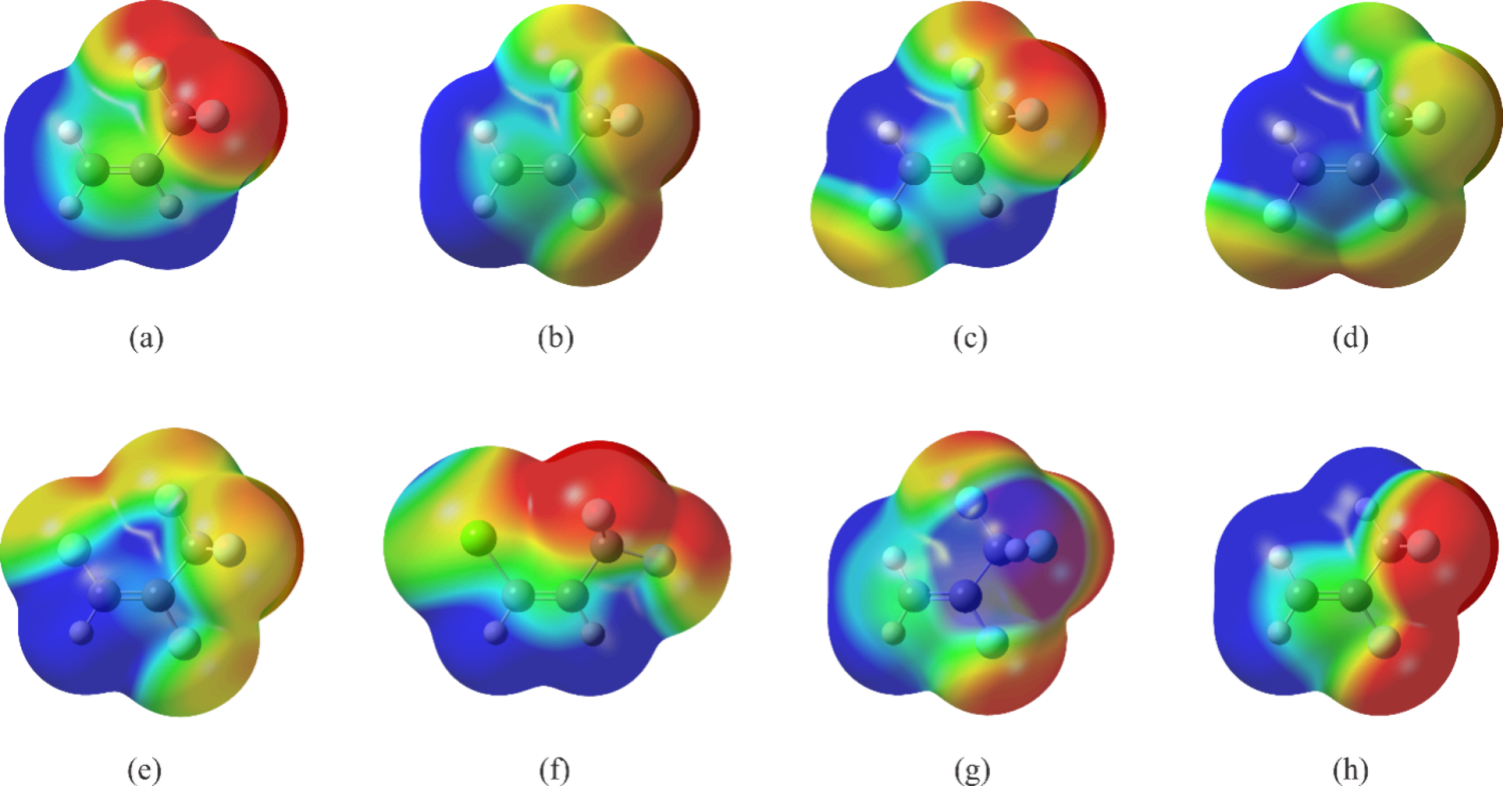
Electrostatic potential mapped onto the total electron density isosurface for (a) TFP, (b) 2,3,3,3-tetrafluoropropene, (c) (*E*)-1,3,3,3-tetrafluoropropene, (d) (*Z*)-1,2,3,3,3-pentafluoropropene, (e) (*E*)-1,2,3,3,3-pentafluoropropene, (f) (*Z*)-1-chloro-3,3,3-trifluoropropene, (g) the lower energy rotamer of 2,3,3-trifluoropropene, and (h) the higher energy rotamer of 2,3,3-trifluoropropene. The same value of electron density and identical color scales are used for the surfaces. Blue represents positive electrostatic potential and red, negative electrostatic potential.

The mapped electrostatic potential surfaces can explain the binding locations for the species we have studied so far very well. In general, there are three motifs for Ar binding to the fluoropropenes: above the planar YCCCF cavity (Y = H or F), above the planar FCCF cavity, or more or less above the C2–F bond. The three complexes with Ar in the YCCCF cavity are Ar-TFP (Figure 8a), Ar-(*E*)-1,3,3,3-tetrafluoropropene (Figure 1b), and Ar-(*E*)-1,2,3,3,3-pentafluoropropene (Figure 1d). This is the cavity that includes C1 and C2 (and by extension, the π electron density in the double bond) and most of the negative region of each of these propenes. In fact, Ar interacts with all the heavy atoms in this cavity. Specifically, judging from the van der Waals contacts for these three complexes (Table 6), the interactions are particularly strong with the in-plane F atom of CF_3_, C1, C3 in all three, and for Ar-(*E*)-1,2,3,3,3-pentafluoropropene, there are additional strong interactions with the F atom *cis* to CF_3_, one of the out-of-plane F atoms of CF_3_, and C2. For (*Z*)-1,2,3,3,3-pentafluoropropene, on the other hand, it is the FCCF cavity that contains most of the electron density and this is where Ar resides, interacting with the atoms forming the cavity and the out-of-plane F atom of CF_3_, with particular strong interactions involving C2, the F atom geminal to CF_3,_ and C1. For 2,3,3,3-tetrafluoropropene (Figure 9b) and the two rotamers of 2,3,3-trifluoropropene (Figure 9g and h), the “right” side of the molecule contains more negative F atoms. As such, Ar binds close to the C2–F bond, interacting particularly strongly with C2, the F atom geminal to the fluoromethyl group, and, except for the lowest energy rotamer of 2,3,3-trifluoropropene, also with the out-of-plane F atom of CF_3_. However, in the complex with this lowest energy rotamer the argon atom also has a significant interaction with C3. Finally, as mentioned earlier, (*Z*)-1-chloro-3,3,3-trifluoropropene contains a CF_3_ group that is arranged differently from the other fluoropropenes. The mapped electrostatic potential surface shows that the F atoms in the CF_3_ group are very negative, likely because they can easily withdraw electron density from the polarizable chlorine atom (Figure 9f). Here, argon is positioned more or less above C1 to interact with C1, C2, one of the out-of-plane F atoms, and the chlorine atom.

**Table 6:** Interaction Lengths in Ångstroms between Ar and Heavy Atoms and Their % Differences from van der Waals Contacts^a^ for Several Halopropenes^b^

	length	% diff	length	%diff	length	% diff	length	% diff
	Ar-TFP		Ar-2,3,3,3-tetrafluoropropene^10^		Ar-(*E*)-1,3,3,3-tetrafluoropropene^11^		Ar-(*Z*)-1,2,3,3,3-pentafluoropropene^12^	
Ar–C1	3.8932(16)	8.7	4.0489(9)	13.1	3.7814(19)	5.6	3.79444(39)	6.0
Ar–C2	4.1202(50)	15.1	3.6592(4)	2.2	4.1068(33)	14.7	3.57091(16)	–0.3
Ar–C3	3.91156(30)	9.3	4.1804(1)	16.8	3.91750(77)	9.4	4.29419(14)	19.9
Ar–F_p_	3.3525(93)	0.1	4.8951(8)	46.1	3.2725(75)	–2.3	4.97297(70)	48.4
Ar–F_o_	3.7267(78)	11.2	3.5358(4)	5.5	3.8322(69)	14.4	3.85307(23)	15.0
Ar–F_g_			3.4615(12)	3.3			3.36875(69)	0.6
Ar–F_c_/Cl_c_								
Ar–F_t_					4.6118(28)	37.7	3.83465(30)	14.5

	Ar-(*E*)-1,2,3,3,3-pentafluoropropene^12^		Ar-(*Z*)-1-chloro-3,3,3-trifluoropropene^13^		Ar-2,3,3-trifluoropropene (rotamer (i))^14^		Ar-2,3,3-trifluoropropene (rotamer (ii))^14^	
Ar–C1	3.73555(15)	4.3	3.6172(58)	1.0	4.0456(18)	13.0	4.2431	18.5
Ar–C2	3.85799(11)	7.8	3.8388(67)	7.2	3.60247(43)	0.6	3.6915	3.1
Ar–C3	3.85190(8)	7.6	4.28922(52)	19.8	3.84539(40)	7.4	4.2942	19.9
Ar–F_p_	3.54731(17)	5.9	5.0145(28)	49.7	4.3644(13)	30.3		
Ar–F_o_	3.57398(15)	6.7	3.6007(60)	7.5			3.6631	9.3
Ar–F_g_	4.71714(21)	40.8			3.6412(13)	8.7	3.1609	–5.6
Ar–F_c_/Cl_c_	3.48774(16)	4.1	3.9044(19)	7.6				
Ar–F_t_								

a The van der Waals contacts are Ar–C, 3.58 Å; Ar–F, 3.35 Å; Ar–Cl, 3.63 Å.

b The F atom on the side of the plane formed by the C atoms opposite that occupied by Ar is too far to interact with it and is not included. The rest of the heavy atoms are F_p_ and F_o_, the F atoms in (or almost in) and away from the plane formed by the C atoms, respectively; F_g_, F_c_, and F_t_ and the F atoms geminal, *cis*, and *trans* to the fluoromethyl group, respectively, and Cl_c_ is the Cl atom *cis* to the fluoromethyl group.

Now we turn to examine the TFP-HCCH complex. The analogous complex, (*Z*)-1-chloro-3,3,3-trifluoropropene, has one more nucleophilic halogen atom (chlorine) and consequently, one less hydrogen atom than TFP. The extra halogen atom helps to withdraw electron density from fewer H atoms, making these atoms more positive than those in TFP. In other words, (*Z*)-1-chloro-3,3,3-trifluoropropene is a better acid than TFP; thus, an interaction between the H atoms located *cis* to each other in TFP and the acetylenic bond of HCCH would not be as stable. Additionally, the CF_3_ group in TFP is arranged so that one of the H atoms in HCCH can interact with the two, very nucleophilic, out-of-plane F atoms while at the same time, the electron rich acetylenic bond can interact with the geminal H atom. It is this configuration, which is also the lowest energy configuration predicted by theory, that we observe. It is worth pointing out that the length of the H-acetylenic bond interaction, 3.0146(81) Å, in HCCH-TFP is similar to the analogous interaction length of 3.0050(10) Å in (*Z*)-1-chloro-3,3,3-trifluoropropene, thus these interactions are likely of similar strength.

This motif adopted by HCCH-TFP is very different from its haloethylene counterpart, namely, HCCH-vinyl fluoride, in which HCCH interacts with the F, H located *cis* to each other.^5^ We have argued that because the H atom involved in the intermolecular interaction is further away from the F atom, it should be less electropositive than the H atom geminal to the F atom. The fact that HCCH does not bind to the geminal F, H pair even though it is electrostatically more favorable is because this configuration is too strained to be stable.^39^ Instead, HCCH binds to the sterically more favorable configuration, the F, H pair across the double bond. For HCCH-TFP, it appears that the binding configuration is driven by electrostatic factors. Not only does HCCH form a bifurcated hydrogen bond with the most negative F atoms, the acetylenic bond also interacts with possibly the most positive H atom in TFP. This is because this H atom is closest to the CF_3_ group, which can withdraw the electron density of that H to a greater extent than those of the other two hydrogen atoms further from the group.

## VI Conclusions

The equilibrium rotational constants provided by theory have guided us well in assigning the rotational spectra of four isotopologues of Ar-TFP and six isotopologues of HCCH-TFP, thus establishing the lowest-energy, average structures of the two complexes. This work allows us to assess the effect on intermolecular interactions of adding a CF_3_ group to ethylene. Interacting via dispersion forces, argon seeks a location that maximizes its interactions with heavy atoms, a common motif observed in haloethylenes and halopropenes. In the present case, it is the planar HCCCF cavity. Turning to electrostatics, in the absence of additional substituents, the F atoms in the CF_3_ group are the most nucleophilic atoms in the molecule, with the two out-of-plane F atoms more so than the in-plane F atom. Thus, in interacting with TFP, HCCH adopts a motif that enables one of its H atoms to interact with this most nucleophilic portion of TFP, forming a bifurcated hydrogen bond. The acetylenic bond, for its part, interacts with the most electropositive portion of TFP. This study forms a foundation for understanding how a protic acid interacts with halopropenes that contain halogen atoms in addition to a CF_3_ group. The motif we have observed in HCCH-TFP is not available to HCCH-(*Z*)-1-chloro-3,3,3-trifluoropropene,^13^ but it is possible, for example, in HCCH-(*E*)-1,3,3,3-tetrafluoropropene. Protic acids such as HF and HCl are stronger gas-phase acids than HCCH and likely also have different steric requirements. A study of additional complexes will help to further understand the nature of intermolecular interactions of halopropenes.

## Supplementary Material



## Notes

Tables of observed and calculated transition frequencies for four isotopologues of 3,3,3-trifluoropropene, four isotopologues of its complex with the argon atom, and six isotopologues of its complex with acetylene that are reported in this study, atomic coordinates for the theoretical structures of the isomers of the argon and acetylene complexes (shown in Figures 1 and 2) and for the experimental structures observed, rotational constants, dipole moment components, and relative equilibrium and zero-point corrected energies for three isomers of Ar-TFP and of Ar–TFP, respectively, obtained from *ab initio* calculations, and the argon-heavy atom distances for the argon complexes; spectroscopic constants obtained for four isotopologues of 3,3,3-trifluoropropene using the Watson *S* reduced and *A* reduced Hamiltonians in the *I* ^*r*^ representation using the transitions obtained in this work and spectroscopic constants for the most abundant isotopologue of Ar-TFP with and without the inclusion of the sextic centrifugal distortion constant *h*_1_ (PDF)
