# Supplementary material for: Microwave Spectra and Molecular Structures of the Gas-Phase Heterodimers Formed between Argon and 3,3,3-Trifluoropropene and between Acetylene and 3,3,3-Trifluoropropene
Source: J Phys Chem A. 2026 Mar 24;130(14):2797–807. doi: 10.1021/acs.jpca.6c00491 (PMC13071910; doi:10.1021/acs.jpca.6c00491)
Supplement: Supplementary file 1 [file jp6c00491_si_001.pdf]

The Microwave Spectra and Molecular Structures of the Gas-Phase Heterodimers Formed Between Argon and 3,3,3-Trifluoropropene and Between Acetylene and 3,3,3-Trifluoropropene

Helen O. Leung*, Mark D. Marshall*, Pohakeaokahokuula G. Mawyer, and Luke N. Kline

Department of Chemistry, Amherst College, P.O. Box 5000, Amherst, MA 01002-5000

Supporting Information

Address for correspondence: Prof. Mark D. Marshall
Department of Chemistry
Amherst College
P.O. Box 5000
Amherst, MA 01002-5000
Telephone: (413) 542-2006
Fax: (413) 542-2735
E-mail: mdmarshall@amherst.edu

*Corresponding authors. Fax: +1-413-542-2735; *e-mail addresses:* hleung@amherst.edu (H.O. Leung), mdmarshall@amherst.edu (M.D. Marshall).

Table S1 presents the atomic coordinates in the appropriate principal inertial axis system for the structures of the argon and acetylene complexes of 3,3,3-trifluoropropene obtained via quantum chemistry calculations. Table S22 contains similar data for the experimentally determined structures along with uncertainties from the structural fits.

Tables S2 through S15 contain the quantum number assignments, observed transition frequencies (in MHz), and the residuals (obs. - calc., also in MHz) from the least squares fits for the isotopologues of 3,3,3-trifluoropropene and its argon and acetylene complexes. The quantum numbers are J, K_a, and K_c, the usual asymmetric top rotational quantum numbers for the initial and final levels of the transition.

Table S16 presents the interaction lengths in Ångstroms between Ar and heavy atoms in three isomers of Ar-TFP obtained from *ab initio* calculations and from experiment.

Tables S17 and S18 present rotational constants, dipole moment components, and relative equilibrium and zero-point corrected energies for three isomers of Ar-TFP and of Ar-HCCH, respectively, obtained from *ab initio* calculations.

Tables S19 and S20 present the spectroscopic constants for four isotopologues of 3,3,3-trifluoropropene determined using the Watson *S* reduced and *A* reduced Hamiltonians, respectively.

Table S21 presents spectroscopic constants for the most abundant isotopologue of Ar-TFP with and without the inclusion of the sextic centrifugal distortion constant h_1.

Table S1: Principal coordinates for the theoretical structures of three isomers each of the argon and acetylene complexes of 3,3,3-trifluoropropene

Ar-3,3,3-trifluoropropene
No BSSE Correction

	Structure (a)			Structure (b)			Structure (c)		
	a	*b*	*c*	*a*	*b*	*c*	*a*	*b*	*c*
C1	-0.4510	2.1651	-0.0221	2.0896	1.9259	0.0000	-3.3367	0.6950	0.0000
C2	-1.0703	1.1690	0.6050	0.9940	1.1718	0.0000	-2.5010	-0.3396	0.0000
C3	-1.2035	-0.1981	0.0276	1.0315	-0.3177	0.0000	-1.0187	-0.1882	0.0000
F1	-0.6398	-0.3248	-1.1814	2.2734	-0.8210	0.0000	-0.6120	1.0886	0.0000
F2	-0.6334	-1.1231	0.8237	0.4048	-0.8229	1.0800	-0.4670	-0.7743	1.0800
F3	-2.4971	-0.5493	-0.1052	0.4048	-0.8229	-1.0800	-0.4670	-0.7743	-1.0800
H	-0.0115	2.0143	-0.9970	3.0703	1.4736	0.0000	-2.9609	1.7075	0.0000
H	-0.3780	3.1411	0.4344	2.0154	3.0034	0.0000	-4.4053	0.5385	0.0000
H	-1.5186	1.2929	1.5808	0.0009	1.5987	0.0000	-2.8505	-1.3625	0.0000
Ar	2.6588	-0.1548	0.0111	-2.8296	0.1847	0.0000	3.0515	0.1462	0.0000

Ar-3,3,3-trifluoropropene
With BSSE Correction

	Structure (a)			Structure (b)			Structure (c)		
	a	*b*	*c*	*a*	*b*	*c*	*a*	*b*	*c*
C1	-0.4390	2.1443	-0.0234	2.0750	1.9645	0.0000	-3.3367	0.6950	0.0000
C2	-1.0859	1.1682	0.6073	1.0063	1.1728	0.0000	-2.5010	-0.3396	0.0000
C3	-1.2711	-0.1921	0.0282	1.0954	-0.3145	0.0000	-1.0187	-0.1882	0.0000
F1	-0.7235	-0.3347	-1.1865	2.3541	-0.7743	0.0000	-0.6120	1.0886	0.0000
F2	-0.7253	-1.1380	0.8168	0.4867	-0.8412	1.0800	-0.4670	-0.7743	1.0800
F3	-2.5771	-0.4986	-0.0931	0.4867	-0.8412	-1.0800	-0.4670	-0.7743	-1.0800
H	-0.0141	1.9811	-1.0028	3.0708	1.5465	0.0000	-2.9609	1.7075	0.0000
H	-0.3285	3.1161	0.4346	1.9634	3.0387	0.0000	-4.4053	0.5385	0.0000
H	-1.5205	1.3048	1.5877	-0.0011	1.5650	0.0000	-2.8505	-1.3625	0.0000
Ar	2.8005	-0.1613	0.0105	-2.9630	0.1652	0.0000	3.0515	0.1462	0.0000

Table S1: Principal coordinates for the theoretical structures of three isomers each of the argon and acetylene complexes of 3,3,3-trifluoropropene

HCCH-3,3,3-trifluoropropene
No BSSE Correction

	Structure (i)			Structure (ii)			Structure (iii)		
	a	*b*	*c*	*a*	*b*	*c*	*a*	*b*	*c*
C1	-1.3195	-2.1340	0.0000	-0.6061	1.5588	0.0000	-0.0866	2.0691	-0.0228
C2	-0.4189	-1.1553	0.0000	-0.2180	0.2867	0.0000	0.7271	1.1875	-0.5970
C3	-0.7864	0.2887	0.0000	1.2133	-0.1274	0.0000	1.0195	-0.1513	-0.0120
F1	-2.1091	0.5034	0.0000	2.0681	0.9045	0.0000	0.3840	-0.3790	1.1455
F2	-0.2876	0.9205	-1.0800	1.5057	-0.8774	1.0800	0.6650	-1.1411	-0.8540
F3	-0.2876	0.9205	1.0800	1.5057	-0.8774	-1.0800	2.3362	-0.3034	0.2281
H	-2.3762	-1.9109	0.0000	0.1213	2.3571	0.0000	-0.5768	1.8397	0.9118
H	-1.0077	-3.1680	0.0000	-1.6556	1.8134	0.0000	-0.2699	3.0289	-0.4827
H	0.6443	-1.3508	0.0000	-0.9249	-0.5312	0.0000	1.2302	1.3917	-1.5318
H	2.2195	1.2395	-0.0001	-3.7936	-0.3125	-1.6617	-1.7943	-0.9502	-0.5612
C	2.8632	0.4004	0.0000	-3.7937	-0.3125	-0.6041	-2.7225	-0.5639	-0.2329
C	3.5987	-0.5583	0.0000	-3.7937	-0.3125	0.6042	-3.7829	-0.1226	0.1422
H	4.2424	-1.3974	0.0001	-3.7937	-0.3125	1.6617	-4.7110	0.2637	0.4705

HCCH-3,3,3-trifluoropropene
With BSSE Correction

	Structure (i)			Structure (ii)			Structure (iii)		
	a	*b*	*c*	*a*	*b*	*c*	*a*	*b*	*c*
C1	-1.2344	-2.1635	0.0824	-0.5849	1.5555	0.0000	0.0373	2.1028	-0.0295
C2	-0.3943	-1.1385	-0.0289	-0.1957	0.2837	0.0000	0.7706	1.1660	-0.6241
C3	-0.8402	0.2832	-0.0239	1.2360	-0.1291	0.0000	1.0397	-0.1663	-0.0135
F1	-2.1664	0.4269	0.1033	2.0898	0.9037	0.0000	0.4720	-0.3271	1.1896
F2	-0.4862	0.9110	-1.1619	1.5291	-0.8788	1.0800	0.5860	-1.1640	-0.7965
F3	-0.2737	0.9702	0.9868	1.5291	-0.8788	-1.0800	2.3610	-0.3732	0.1467
H	-2.2968	-1.9974	0.1829	0.1417	2.3545	0.0000	-0.4003	1.9264	0.9420
H	-0.8668	-3.1790	0.0740	-1.6347	1.8091	0.0000	-0.1320	3.0554	-0.5094
H	0.6730	-1.2768	-0.1306	-0.9018	-0.5348	0.0000	1.2199	1.3166	-1.5957
H	2.3524	1.1493	0.5811	-3.8767	-0.3058	-1.6617	-1.9450	-0.9867	-0.5530

Table S1: Principal coordinates for the theoretical structures of three isomers each of the argon and acetylene complexes of 3,3,3-trifluoropropene

C	3.0014	0.3905	0.2328	-3.8767	-0.3058	-0.6041	-2.8555	-0.5584	-0.2274
C	3.7430	-0.4766	-0.1652	-3.8767	-0.3058	0.6042	-3.8957	-0.0689	0.1445
H	4.3920	-1.2355	-0.5135	-3.8767	-0.3058	1.6617	-4.8062	0.3595	0.4700

Table S2: Observed transition frequencies (in MHz) for CH_2CHCF_3

J'	K_a'	K_c'	J''	K_a''	K_c''	Observed	Obs - Calc
8	1	7	8	1	8	2522.6628	0.0023
1	1	0	1	0	1	2569.5404	0.0047
2	1	1	2	0	2	2641.2907	0.0017
3	1	2	3	0	3	2751.6493	0.0005
4	1	3	4	0	4	2903.8389	-0.0006
5	1	4	5	0	5	3102.0390	0.0020
9	1	8	9	1	9	3146.8091	-0.0020
6	1	5	6	0	6	3351.1926	0.0010
7	1	6	7	0	7	3656.7330	0.0019
10	1	9	10	1	10	3835.1293	-0.0049
8	1	7	8	0	8	4024.1265	0.0025
9	1	8	9	0	9	4458.3105	-0.0069
11	1	10	11	1	11	4584.6583	-0.0020
10	1	9	10	0	10	4963.1082	0.0029
12	1	11	12	1	12	5391.5469	0.0052
11	1	10	11	0	11	5540.5345	0.0028
1	0	1	0	0	0	5816.6361	0.0014
12	1	11	12	0	12	6190.4419	0.0021
12	2	10	12	1	11	6353.2530	-0.0018
11	2	9	11	1	10	6370.3728	0.0035
13	2	11	13	1	12	6392.0255	0.0074
10	2	8	10	1	9	6435.1666	0.0035
14	2	12	14	1	13	6493.8730	0.0051
9	2	7	9	1	8	6538.4558	0.0040
15	2	13	15	1	14	6665.1165	0.0027
8	2	6	8	1	7	6670.2377	0.0019
7	2	5	7	1	6	6820.0500	0.0009
13	1	12	13	0	13	6910.2596	0.0054
6	2	4	6	1	5	6977.4293	0.0012
5	2	3	5	1	4	7132.4106	0.0032
4	2	2	4	1	3	7275.9588	0.0030
3	2	1	3	1	2	7400.3005	0.0031
2	2	0	2	1	1	7499.1101	-0.0017
14	1	13	14	0	14	7695.0176	-0.0062
2	2	1	2	1	2	7708.5971	0.0021
3	2	2	3	1	3	7814.8581	0.0005
4	2	3	4	1	4	7957.0652	0.0009
5	2	4	5	1	5	8135.6084	0.0009
1	1	1	0	0	0	8315.8575	0.0027
6	2	5	6	1	6	8350.9120	0.0015
7	2	6	7	1	7	8603.3880	0.0029
8	2	7	8	1	8	8893.3905	0.0052
2	0	2	1	1	1	9132.5786	0.0020

Table S2: Observed transition frequencies (in MHz) for CH_2CHCF_3

J'	K_a'	K_c'	J''	K_a''	K_c''	Observed	Obs - Calc
9	2	8	9	1	9	9221.1618	0.0030
16	1	15	16	0	16	9429.5981	0.0005
10	2	9	10	1	10	9586.8020	0.0036
3	1	3	2	2	0	9633.3826	-0.0053
11	2	10	11	1	11	9990.2028	0.0053
3	1	2	2	2	1	10056.7242	-0.0044
12	2	11	12	1	12	10431.0181	0.0068
4	2	3	3	3	0	10592.2538	-0.0165
13	2	12	13	1	13	10908.6192	-0.0093
14	3	11	14	2	12	11323.2902	-0.0055
2	1	2	1	1	1	11562.9188	0.0005
13	3	10	13	2	11	11585.2925	0.0075
2	0	2	1	0	1	11631.7942	-0.0024
2	1	1	1	1	0	11703.5486	-0.0014
12	3	9	12	2	10	11823.9500	-0.0002
11	3	8	11	2	9	12033.2277	0.0084
10	3	7	10	2	8	12209.5706	0.0062
9	3	6	9	2	7	12352.0102	-0.0059
8	3	5	8	2	6	12461.9298	0.0083
7	3	4	7	2	5	12542.4731	-0.0028
6	3	3	6	2	4	12598.1083	-0.0035
5	3	2	5	2	3	12633.8553	-0.0038
4	3	1	4	2	2	12654.7738	-0.0016
3	3	0	3	2	1	12665.4867	-0.0186
3	3	1	3	2	2	12672.8095	0.0069
4	3	2	4	2	3	12676.5545	-0.0074
5	3	3	5	2	4	12684.3295	-0.0090
6	3	4	6	2	5	12698.0761	-0.0052
7	3	5	7	2	6	12720.0238	-0.0042
8	3	6	8	2	7	12752.6433	-0.0090
9	3	7	9	2	8	12798.5994	-0.0072
10	3	8	10	2	9	12860.6513	-0.0078
11	3	9	11	2	10	12941.6235	-0.0060
12	3	10	12	2	11	13044.3164	-0.0079
13	3	11	13	2	12	13171.4682	-0.0064
2	1	2	1	0	1	14062.1415	0.0032
3	0	3	2	1	2	15013.6724	-0.0024
4	1	4	3	2	1	15300.7252	0.0138
4	1	3	3	2	2	16011.0565	0.0002
5	2	4	4	3	1	16402.7603	-0.0012
5	2	3	4	3	2	16453.9113	0.0027
3	1	3	2	1	2	17343.4469	0.0000
3	0	3	2	0	2	17444.0171	0.0006

Table S2: Observed transition frequencies (in MHz) for CH_2CHCF_3

J'	K_a'	K_c'	J''	K_a''	K_c''	Observed	Obs - Calc
3	2	2	2	2	1	17449.7155	0.0059
3	2	1	2	2	0	17455.5633	0.0014
3	1	2	2	1	1	17554.3787	0.0024
10	4	6	10	3	7	17676.1325	0.0050
9	4	5	9	3	6	17698.7405	0.0029
8	4	4	8	3	5	17714.3128	0.0082
9	4	6	9	3	7	17715.9923	0.0070
8	4	5	8	3	6	17722.9685	-0.0141
7	4	3	7	3	4	17724.6974	-0.0083
7	4	4	7	3	5	17728.6639	-0.0060
6	4	2	6	3	3	17731.4265	0.0135
5	4	1	5	3	2	17735.5648	0.0093

Table S3: Observed transition frequencies (in MHz) for $^{13}CH_2CHCF_3$

J'	K_a'	K_c'	J''	K_a''	K_c''	Observed	Obs - Calc
1	1	0	1	0	1	2645.9350	0.0000
2	1	1	2	0	2	2713.7447	-0.0018
3	1	2	3	0	3	2817.8381	-0.0002
4	1	3	4	0	4	2961.0140	-0.0060
5	1	4	5	0	5	3146.9514	0.0063
6	1	5	6	0	6	3379.9867	0.0100
7	1	6	7	0	7	3664.9670	0.0035
1	0	1	0	0	0	5660.3416	0.0014
8	2	6	8	1	7	6929.6348	-0.0056
6	2	4	6	1	5	7236.9004	0.0121
5	2	3	5	1	4	7387.9016	-0.0027
4	2	2	4	1	3	7526.3069	0.0041
3	2	1	3	1	2	7645.2983	0.0070
2	2	0	2	1	1	7739.3655	-0.0003
2	2	1	2	1	2	7937.7850	-0.0037
3	2	2	3	1	3	8038.3043	-0.0024
4	2	3	4	1	4	8172.7861	-0.0023
1	1	1	0	0	0	8239.7100	-0.0001
5	2	4	5	1	5	8341.5780	-0.0032
6	2	5	6	1	6	8545.0554	-0.0097
2	0	2	1	1	1	8740.0399	0.0078
8	2	7	8	1	8	9057.5843	0.0021
2	1	2	1	1	1	11254.0855	0.0027
2	0	2	1	0	1	11319.3930	-0.0090
2	1	1	1	1	0	11387.2128	-0.0007
5	3	2	5	2	3	13030.0448	-0.0012
5	3	3	5	2	4	13074.0090	0.0030
6	3	4	6	2	5	13085.9394	-0.0008
2	1	2	1	0	1	13833.4509	-0.0018
3	0	3	2	1	2	14461.8512	-0.0077
3	1	3	2	1	2	16880.3316	0.0121
3	0	3	2	0	2	16975.9116	0.0020
3	2	2	2	2	1	16980.8386	0.0009
3	2	1	2	2	0	16985.9237	-0.0031
3	1	2	2	1	1	17079.9974	-0.0039

Table S4: Observed transition frequencies (in MHz) for $CH_2{}^{13}CHCF_3$

J'	K_a'	K_c'	J''	K_a''	K_c''	Observed	Obs - Calc
1	1	0	1	0	1	2568.1737	0.0103
2	1	1	2	0	2	2646.6741	0.0031
3	1	2	3	0	3	2767.6930	0.0022
4	1	3	4	0	4	2935.0606	-0.0028
6	1	5	6	0	6	3429.5420	0.0119
7	1	6	7	0	7	3768.6957	-0.0079
1	0	1	0	0	0	5772.1392	-0.0016
7	2	5	7	1	6	6754.6492	-0.0003
6	2	4	6	1	5	6917.6546	0.0001
4	2	2	4	1	3	7234.6056	-0.0038
3	2	1	3	1	2	7368.6805	-0.0009
2	2	1	2	1	2	7704.4789	0.0038
3	2	2	3	1	3	7820.6224	0.0009
4	2	3	4	1	4	7976.0978	-0.0060
5	2	4	5	1	5	8171.3852	0.0042
1	1	1	0	0	0	8263.5200	-0.0007
6	2	5	6	1	6	8406.9346	-0.0045
2	0	2	1	1	1	9051.1473	0.0040
3	1	2	2	2	1	9956.2945	0.0041
2	1	2	1	1	1	11467.4610	-0.0022
2	0	2	1	0	1	11542.5232	0.0000
2	1	1	1	1	0	11621.0326	0.0019
6	3	3	6	2	4	12561.2573	-0.0038
5	3	2	5	2	3	12603.6556	0.0131
4	3	1	4	2	2	12628.4745	-0.0029
4	3	2	4	2	3	12654.4700	-0.0049
5	3	3	5	2	4	12663.8227	0.0006
2	1	2	1	0	1	13958.8414	-0.0018
3	0	3	2	1	2	14893.0644	-0.0103
3	1	3	2	1	2	17200.0857	-0.0016
3	0	3	2	0	2	17309.3927	-0.0020
3	2	2	2	2	1	17316.2419	0.0081
3	2	1	2	2	0	17323.2226	-0.0005
3	1	2	2	1	1	17430.4162	0.0017

Table S5: Observed transition frequencies (in MHz) for $CH_2CH^{13}CF_3$

J'	K_a'	K_c'	J''	K_a''	K_c''	Observed	Obs - Calc
1	1	0	1	0	1	2571.1681	0.0040
2	1	1	2	0	2	2642.8722	-0.0007
3	1	2	3	0	3	2753.1607	0.0000
5	1	4	5	0	5	3103.2948	-0.0034
6	1	5	6	0	6	3352.2570	-0.0015
1	0	1	0	0	0	5814.3902	-0.0005
8	2	6	8	1	7	6675.3511	0.0055
7	2	5	7	1	6	6825.2113	-0.0110
6	2	4	6	1	5	6982.6160	0.0032
5	2	3	5	1	4	7137.5651	-0.0022
4	2	2	4	1	3	7281.0660	-0.0033
3	2	1	3	1	2	7405.3548	-0.0017
2	2	1	2	1	2	7713.4824	0.0027
3	2	2	3	1	3	7819.6765	-0.0013
4	2	3	4	1	4	7961.7969	-0.0007
5	2	4	5	1	5	8140.2267	-0.0039
1	1	1	0	0	0	8315.2844	0.0033
6	2	5	6	1	6	8355.3947	-0.0049
7	2	6	7	1	7	8607.7222	0.0067
2	0	2	1	1	1	9126.4200	0.0010
3	1	3	2	2	0	9621.8313	-0.0008
3	1	2	2	2	1	10044.9094	-0.0088
2	1	2	1	1	1	11558.4684	-0.0022
2	0	2	1	0	1	11627.3095	0.0000
2	1	1	1	1	0	11699.0169	-0.0014
6	3	3	6	2	4	12606.4890	0.0008
4	3	1	4	2	2	12663.0349	-0.0161
4	3	2	4	2	3	12684.8097	0.0115
5	3	3	5	2	4	12692.5643	0.0027
6	3	4	6	2	5	12706.2822	0.0014
2	1	2	1	0	1	14059.3613	0.0002
3	0	3	2	1	2	15005.2356	-0.0016
3	1	3	2	1	2	17336.7737	0.0005
3	0	3	2	0	2	17437.2891	0.0004
3	2	2	2	2	1	17442.9787	0.0073
3	2	1	2	2	0	17448.8154	0.0022
3	1	2	2	1	1	17547.5778	0.0013

Table S6: Observed transition frequencies (in MHz) for Ar-CH_2CHCF_3

J'	K_a'	K_c'	J''	K_a''	K_c''	Observed	Obs - Calc
5	1	5	4	2	2	2002.7656	-0.0002
1	1	1	1	0	1	2011.7658	0.0052
1	1	0	1	0	1	2137.6022	0.0016
2	1	1	2	0	2	2269.0989	-0.0005
9	3	7	8	4	4	2295.7242	0.0041
9	3	6	8	4	5	2441.9889	0.0167
7	2	6	6	3	3	2469.6429	-0.0013
3	1	2	3	0	3	2476.8854	0.0004
7	2	6	6	3	4	2483.1897	0.0073
4	1	3	4	0	4	2772.9182	0.0007
3	3	1	4	2	2	2865.7965	-0.0007
3	3	0	4	2	3	2950.9362	0.0047
7	2	5	6	3	3	3140.2445	-0.0070
7	2	5	6	3	4	3153.7933	0.0036
5	1	4	5	0	5	3171.3786	-0.0018
3	0	3	2	1	1	3281.4909	-0.0053
5	4	2	6	3	3	3322.1469	-0.0085
5	4	1	6	3	4	3335.7323	0.0044
6	1	6	5	2	3	3343.3589	0.0003
6	1	6	5	2	4	3539.1895	-0.0046
2	1	2	1	1	1	3589.8113	-0.0013
9	2	8	9	1	8	3649.9545	0.0071
3	0	3	2	1	2	3658.9974	-0.0008
6	1	5	6	0	6	3686.2457	-0.0006
2	0	2	1	0	1	3709.9754	-0.0005
2	1	1	1	1	0	3841.4731	-0.0015
1	1	1	0	0	0	3869.6391	0.0007
12	4	8	11	5	7	3870.4268	-0.0064
5	1	4	4	2	2	3885.2082	0.0013
5	1	4	4	2	3	3970.1773	-0.0015
1	1	0	0	0	0	3995.4785	0.0001
8	2	7	8	1	7	4115.0500	0.0033
10	3	8	9	4	5	4167.9386	-0.0014
8	2	7	7	3	4	4199.5096	-0.0011
8	2	7	7	3	5	4233.1298	-0.0022
7	1	6	7	0	7	4327.9172	-0.0008
10	3	7	9	4	6	4435.5886	0.0029
7	1	7	6	2	4	4532.8061	-0.0005
7	2	6	7	1	6	4552.5766	0.0012
4	0	4	3	1	2	4897.5441	-0.0010
7	1	7	6	2	5	4916.6330	0.0024
6	2	5	6	1	5	4951.8614	0.0014
8	1	7	8	0	8	5099.8634	-0.0003

Table S6: Observed transition frequencies (in MHz) for Ar-CH_2CHCF_3

J'	K_a'	K_c'	J''	K_a''	K_c''	Observed	Obs - Calc
8	2	6	8	1	7	5188.7492	0.0002
4	4	1	5	3	2	5203.1809	-0.0102
4	4	0	5	3	3	5207.7230	-0.0037
7	2	5	7	1	6	5223.1839	0.0011
9	2	7	9	1	8	5254.4115	0.0017
5	2	4	5	1	4	5304.8119	0.0018
8	2	6	7	3	5	5306.8348	0.0006
6	2	4	6	1	5	5335.6859	0.0020
3	1	3	2	1	2	5381.0964	0.0011
10	2	8	10	1	9	5438.3654	-0.0006
5	2	3	5	1	4	5500.6464	0.0008
8	1	8	7	2	5	5549.0455	0.0017
3	0	3	2	0	2	5550.5960	0.0005
3	2	2	2	2	1	5573.0836	-0.0009
3	2	1	2	2	0	5595.8759	-0.0003
2	1	2	1	0	1	5601.5740	0.0008
4	2	3	4	1	3	5605.5477	0.0001
4	0	4	3	1	3	5652.3311	-0.0018
4	2	2	4	1	3	5690.5203	0.0009
11	2	9	11	1	10	5755.4627	0.0004
3	1	2	2	1	1	5758.3807	-0.0005
13	4	9	12	5	8	5821.4838	-0.0059
3	2	2	3	1	2	5849.9567	0.0020
9	2	8	8	3	5	5865.5122	-0.0014
3	2	1	3	1	2	5878.4663	0.0016
6	1	5	5	2	3	5971.2054	-0.0002
2	1	1	1	0	1	5979.0759	0.0007
9	1	8	9	0	9	5996.5221	-0.0013
11	3	9	10	4	6	6027.9635	-0.0016
2	2	1	2	1	1	6035.2542	0.0028
2	2	0	2	1	1	6040.9721	0.0025
6	1	5	5	2	4	6167.0378	-0.0032
12	2	10	12	1	11	6217.2422	0.0011
8	1	8	7	2	6	6219.6450	-0.0062
9	1	9	8	2	6	6375.2448	-0.0001
5	0	5	4	1	3	6404.3469	0.0010
2	2	1	2	1	2	6412.7550	0.0017
2	2	0	2	1	2	6418.4737	0.0021
11	3	8	10	4	7	6486.7155	-0.0044
3	2	2	3	1	3	6604.7440	0.0015
3	2	1	3	1	3	6633.2537	0.0012
13	2	11	13	1	12	6830.9046	0.0025
4	2	3	4	1	4	6862.5431	0.0001

Table S6: Observed transition frequencies (in MHz) for Ar-CH_2CHCF_3

J'	K_a'	K_c'	J''	K_a''	K_c''	Observed	Obs - Calc
4	2	2	4	1	4	6947.5155	0.0007
10	1	10	9	2	7	7001.9809	0.0011
10	1	9	10	0	10	7003.2681	-0.0011
4	1	4	3	1	3	7168.2525	-0.0024
5	2	4	5	1	5	7187.2509	-0.0004
3	1	3	2	0	2	7272.6928	0.0001
4	0	4	3	0	3	7374.4293	-0.0007
5	2	3	5	1	5	7383.0873	0.0006
4	2	3	3	2	2	7426.0538	-0.0016
11	1	11	10	2	8	7427.6012	0.0016
4	3	2	3	3	1	7440.9283	-0.0078
4	3	1	3	3	0	7441.9043	-0.0050
10	2	9	9	3	6	7447.7040	0.0007
9	2	7	8	3	5	7469.9832	0.0072
4	2	2	3	2	1	7482.5153	-0.0019
9	2	7	8	3	6	7543.2116	0.0021
6	2	5	6	1	6	7579.7087	0.0018
14	2	12	14	1	13	7597.4062	-0.0012
5	0	5	4	1	4	7661.3411	-0.0003
4	1	3	3	1	2	7670.4627	0.0002
6	0	6	5	1	4	7785.6035	-0.0014
12	3	10	11	4	7	7866.4971	-0.0042
15	3	12	15	2	13	7872.2072	0.0038
14	3	11	14	2	12	7937.1832	-0.0030
16	3	13	16	2	14	7943.6800	-0.0059
6	2	4	6	1	6	7963.5349	0.0041
7	1	6	6	2	4	8020.4415	-0.0009
3	1	2	2	0	2	8027.4814	0.0009
7	2	6	7	1	7	8040.2119	0.0007
11	1	10	11	0	11	8098.2716	0.0009
13	3	10	13	2	11	8115.9242	-0.0015
17	3	14	17	2	15	8169.2052	0.0002
11	3	9	11	2	9	8252.7410	-0.0109
12	3	9	12	2	10	8380.7531	-0.0014
7	1	6	6	2	5	8404.2654	-0.0009
15	2	13	15	1	14	8509.6936	0.0054
18	3	15	18	2	16	8561.3107	0.0008
8	2	7	8	1	8	8568.2661	-0.0004
10	1	10	9	2	8	8606.4325	-0.0097
12	3	9	11	4	8	8609.8589	-0.0003
11	3	8	11	2	9	8700.2572	-0.0014
7	2	5	7	1	7	8710.8165	-0.0021
10	3	8	10	2	8	8778.9048	0.0001

Table S6: Observed transition frequencies (in MHz) for Ar-CH_2CHCF_3

J'	K_a'	K_c'	J''	K_a''	K_c''	Observed	Obs - Calc
4	1	4	3	0	3	8890.3552	0.0031
11	2	10	10	3	7	8923.0950	0.0026
5	1	5	4	1	4	8950.2825	0.0019
7	0	7	6	1	5	9028.2113	0.0030
10	3	7	10	2	8	9041.6984	0.0018
9	2	8	9	1	9	9162.3730	-0.0012
5	0	5	4	0	4	9177.2618	-0.0016
11	2	10	10	3	8	9185.8828	-0.0016
9	3	7	9	2	7	9229.8270	0.0034
12	1	11	12	0	12	9255.5898	0.0016
5	2	4	4	2	3	9274.9909	0.0021
5	4	1	4	4	0	9298.8160	0.0009
5	4	2	4	4	1		
5	3	3	4	3	2	9305.0167	0.0018
5	3	2	4	3	1	9308.4096	-0.0014
9	3	6	9	2	7	9374.2003	0.0008
5	2	3	4	2	2	9385.8541	0.0016
16	2	14	16	1	15	9552.0121	0.0040
5	1	4	4	1	3	9575.7269	0.0006
8	3	6	8	2	6	9598.8732	0.0012
8	2	6	8	1	8	9641.9668	-0.0020
6	0	6	5	1	5	9668.0450	-0.0010
13	3	11	12	4	8	9671.5703	-0.0002
8	3	5	8	2	6	9672.1067	0.0012
11	1	11	10	2	9	9695.3304	0.0004
10	2	8	9	3	6	9715.4337	0.0001
10	2	9	10	1	10	9819.9217	-0.0013
15	4	11	14	5	10	9827.4110	-0.0019
10	2	8	9	3	7	9859.8148	0.0053
2	2	1	1	1	0	9876.7274	0.0013
2	2	0	1	1	0	9882.4488	0.0045
7	3	5	7	2	5	9884.1784	0.0000
7	3	4	7	2	5	9917.7996	-0.0002
8	1	7	7	2	5	10002.2589	-0.0047
2	2	1	1	1	1	10002.5714	0.0054
2	2	0	1	1	1	10008.2893	0.0051
6	3	4	6	2	4	10089.8384	0.0030
6	3	3	6	2	4	10103.3758	0.0022
8	0	8	7	1	6	10125.5814	-0.0013
4	1	3	3	0	3	10147.3496	0.0021
5	3	3	5	2	3	10225.8940	-0.0018
5	3	2	5	2	3	10230.4281	0.0006
12	2	11	11	3	8	10266.4629	0.0080

Table S6: Observed transition frequencies (in MHz) for Ar-CH_2CHCF_3

J'	K_a'	K_c'	J''	K_a''	K_c''	Observed	Obs - Calc
4	3	2	4	2	2	10306.7335	0.0002
4	3	1	4	2	2	10307.8685	-0.0006
3	3	1	3	2	1	10348.3138	-0.0006
3	3	0	3	2	1	10348.4812	0.0043
3	3	1	3	2	2	10376.8235	-0.0010
3	3	0	3	2	2	10376.9914	0.0045
4	3	2	4	2	3	10391.7069	0.0018
4	3	1	4	2	3	10392.8424	0.0015
5	3	3	5	2	4	10421.7295	-0.0016
5	3	2	5	2	4	10426.2609	-0.0020
13	1	12	13	0	13	10448.7730	-0.0090
5	1	5	4	0	4	10466.2068	0.0042
6	3	4	6	2	5	10473.6586	-0.0007
6	3	3	6	2	5	10487.1978	0.0002
11	2	10	11	1	11	10537.1854	-0.0041
7	3	5	7	2	6	10554.7854	-0.0003
7	3	4	7	2	6	10588.4052	-0.0019
8	3	6	8	2	7	10672.5725	-0.0017
8	1	7	7	2	6	10672.8667	-0.0043
6	1	6	5	1	5	10726.4448	-0.0005
8	3	5	8	2	7	10745.8104	0.0027
9	2	7	9	1	9	10766.8448	0.0083
13	3	10	12	4	9	10818.1101	0.0030
9	3	7	9	2	8	10834.2857	-0.0004
6	0	6	5	0	5	10956.9852	-0.0001
10	3	8	10	2	9	11046.6368	0.0017
9	0	9	8	1	7	11079.8887	-0.0085
6	2	5	5	2	4	11118.8974	-0.0036
6	4	3	5	4	2	11162.0555	0.0076
6	4	2	5	4	1	11162.1872	0.0021
6	3	4	5	3	3	11170.8271	-0.0020
6	3	3	5	3	2	11179.8313	-0.0043
6	2	4	5	2	3	11306.8899	0.0004
12	2	11	12	1	12	11309.4495	-0.0003
11	3	9	11	2	10	11315.4736	-0.0111
13	2	12	12	3	9	11451.9607	0.0033
6	1	5	5	1	4	11471.8521	0.0010
3	2	2	2	1	1	11608.3368	0.0010
3	2	1	2	1	1	11636.8475	0.0017
12	3	10	12	2	11	11645.6005	0.0027
14	1	13	14	0	14	11654.3593	0.0013
7	0	7	6	1	6	11656.0558	0.0006
9	1	8	8	2	6	11887.6743	0.0026

Table S6: Observed transition frequencies (in MHz) for Ar-CH_2CHCF_3

J'	K_a'	K_c'	J''	K_a''	K_c''	Observed	Obs - Calc
10	0	10	9	1	8	11902.4042	-0.0036
16	4	12	15	5	11	11903.9624	-0.0024
3	2	2	2	1	2	11985.8386	0.0008
3	2	1	2	1	2	12014.3495	0.0016
6	1	6	5	0	5	12015.3860	0.0015
13	3	11	13	2	12	12040.4375	-0.0053
13	2	12	13	1	13	12131.1823	-0.0082
11	2	9	10	3	8	12248.6168	-0.0004
5	1	4	4	0	4	12348.6442	0.0004
7	1	7	6	1	6	12496.3354	-0.0020
14	3	12	14	2	13	12502.0590	0.0009
11	0	11	10	1	9	12612.1445	-0.0104
7	0	7	6	0	6	12714.4560	0.0015
15	4	11	15	3	12	12751.7900	0.0030
15	1	14	15	0	15	12854.2965	-0.0003
7	2	6	6	2	5	12956.8436	0.0019
9	1	8	8	2	7	12961.3720	-0.0020
14	2	13	14	1	14	12996.3848	-0.0005
7	5	2	6	5	1	13017.5090	0.0078
7	5	3	6	5	2		
7	4	4	6	4	3	13027.1333	0.0065
7	4	3	6	4	2	13027.5800	-0.0029
15	3	13	15	2	14	13030.9685	-0.0109
7	3	5	6	3	4	13037.9651	-0.0030
7	3	4	6	3	3	13058.0476	-0.0037
15	3	13	14	4	10	13118.6309	0.0011
14	4	10	14	3	11	13170.4339	0.0027
7	2	5	6	2	4	13243.6281	0.0030
4	2	3	3	1	2	13276.0085	-0.0016
7	1	6	6	1	5	13356.1287	0.0024
4	2	2	3	1	2	13360.9850	0.0031
13	4	9	13	3	10	13529.3941	0.0025
7	1	7	6	0	6	13554.7386	0.0019
8	0	8	7	1	7	13613.2175	-0.0009
16	3	14	16	2	15	13626.2363	0.0013
10	1	9	9	2	7	13651.2736	0.0064
12	4	8	12	3	9	13820.8313	0.0016
15	2	14	15	1	15	13898.8010	0.0004
4	2	3	3	1	3	14030.7997	0.0018
11	4	7	11	3	8	14045.5558	0.0044
4	2	2	3	1	3	14115.7725	0.0028
10	4	6	10	3	7	14210.6109	-0.0011
8	1	8	7	1	7	14259.8597	-0.0027

Table S6: Observed transition frequencies (in MHz) for Ar-CH_2CHCF_3

J'	K_a'	K_c'	J''	K_a''	K_c''	Observed	Obs - Calc
9	4	5	9	3	6	14326.3993	0.0010
8	4	4	8	3	5	14404.0785	-0.0012
7	4	4	7	3	4	14453.2779	-0.0009
8	0	8	7	0	7	14453.5001	-0.0005
7	4	3	7	3	4	14453.9053	-0.0012
10	4	7	10	3	8	14462.1570	0.0010
9	4	6	9	3	7	14465.9249	0.0044
11	4	8	11	3	9	14469.1719	-0.0004
10	4	6	10	3	8	14473.3967	-0.0073
8	4	5	8	3	6	14475.4340	-0.0029
8	4	4	8	3	6	14477.3196	0.0065
6	4	3	6	3	3	14484.1967	-0.0065
6	4	2	6	3	3	14484.3743	-0.0005
7	4	4	7	3	5	14486.8945	-0.0057
7	4	3	7	3	5	14487.5299	0.0021
12	4	9	12	3	10	14493.0977	0.0092
6	4	3	6	3	4	14497.7451	0.0037
6	4	2	6	3	4	14497.9134	0.0003
5	4	1	5	3	2	14502.0277	0.0024
5	4	2	5	3	3	14506.5219	-0.0008
4	4	0	4	3	1	14511.6023	-0.0035
4	4	1	4	3	2	14512.7297	-0.0081
13	4	10	13	3	11	14540.8729	0.0019
14	4	11	14	3	12	14620.0033	-0.0013
6	1	5	5	0	5	14643.2305	-0.0011
15	4	12	15	3	13	14738.1206	0.0037
5	2	4	4	1	3	14880.5355	-0.0010
8	3	6	7	3	5	14905.7096	0.0035
5	2	3	4	1	3	15076.3707	-0.0012
8	1	8	7	0	7	15100.1429	-0.0017
8	2	6	7	2	5	15191.0071	-0.0055
8	1	7	7	1	6	15225.4460	-0.0003
10	1	9	9	2	8	15255.7280	-0.0016
11	1	10	10	2	8	15272.0730	0.0134
9	0	9	8	1	8	15533.1122	-0.0048
3	3	1	2	2	0	15944.1908	0.0001
3	3	0	2	2	1	15950.0733	0.0019
9	1	9	8	1	8	16017.2104	-0.0033
5	2	4	4	1	4	16137.5314	-0.0004
9	0	9	8	0	8	16179.7580	-0.0030
5	2	3	4	1	4	16333.3650	-0.0022
6	2	5	5	1	4	16423.7113	0.0002
9	2	8	8	2	7	16611.3177	-0.0036

Table S6: Observed transition frequencies (in MHz) for Ar-CH_2CHCF_3

J'	K_a'	K_c'	J''	K_a''	K_c''	Observed	Obs - Calc
9	1	9	8	0	8	16663.8558	-0.0018
12	1	11	11	2	9	16733.7954	0.0076
9	4	6	8	4	5	16763.5154	-0.0014
9	4	5	8	4	4	16766.4898	-0.0044
6	2	4	5	1	4	16807.5359	0.0009
9	3	6	8	3	5	16844.1696	-0.0060
7	1	6	6	0	6	17042.3625	-0.0100
9	1	8	8	1	7	17076.4220	0.0013
9	2	7	8	2	6	17142.0864	0.0049
13	2	11	12	3	10	17195.2781	0.0081
10	0	10	9	1	9	17414.8356	0.0010
11	1	10	10	2	9	17539.7923	0.0023
10	1	10	9	1	9	17768.8157	-0.0007
4	3	2	3	2	1	17789.2465	-0.0039
4	3	1	3	2	1	17790.3873	0.0010
4	3	2	3	2	2	17817.7657	0.0052
4	3	1	3	2	2	17818.8996	0.0033
10	0	10	9	0	9	17898.9230	-0.0083
7	2	6	6	1	5	17908.7036	0.0019

Table S7: Observed transition frequencies (in MHz) for Ar-$^{13}CH_2CHCF_3$

J'	K_a'	K_c'	J''	K_a''	K_c''	Observed	Obs - Calc
2	1	1	2	0	2	2203.9286	0.0030
3	1	2	3	0	3	2424.7911	0.0056
4	1	3	4	0	4	2740.9846	0.0008
5	1	4	5	0	5	3168.3489	0.0000
3	0	3	2	1	2	3719.0901	0.0007
6	1	5	6	0	6	3721.7304	0.0046
1	1	1	0	0	0	3782.6929	-0.0075
5	1	4	4	2	3	4207.0569	-0.0029
8	2	6	8	1	7	5008.2316	-0.0013
7	2	5	7	1	6	5008.5762	0.0009
6	2	4	6	1	5	5099.6247	0.0046
4	2	2	4	1	3	5442.5329	-0.0029
3	2	1	3	1	2	5634.3549	-0.0040
4	0	4	3	1	3	5707.3225	-0.0015
3	2	2	3	1	3	6396.9395	-0.0087
4	2	3	4	1	4	6669.2652	0.0029
5	2	4	5	1	5	7012.3835	-0.0029
3	1	3	2	0	2	7154.5100	0.0032
5	0	5	4	1	4	7707.9827	0.0095
7	1	6	6	2	5	8654.0293	-0.0051
4	1	4	3	0	3	8753.1342	0.0043
8	3	5	8	2	6	9210.5218	-0.0048
7	3	4	7	2	5	9478.8091	0.0033
2	2	1	1	1	0	9630.0215	0.0026
6	3	3	6	2	4	9685.3684	-0.0033
6	0	6	5	1	5	9701.3346	0.0021
2	2	0	1	1	1	9769.2373	0.0021
5	3	2	5	2	3	9828.8115	0.0049
4	3	1	4	2	2	9917.0653	0.0076
3	3	1	3	2	2	9996.2198	-0.0067
4	3	2	4	2	3	10013.4213	0.0015
5	3	3	5	2	4	10047.9680	-0.0042
6	3	4	6	2	5	10107.5260	0.0122
7	3	5	7	2	6	10200.1883	-0.0039
5	1	5	4	0	4	10309.8053	0.0034
8	3	6	8	2	7	10334.2229	0.0015
3	2	2	2	1	1	11347.5267	-0.0027
3	2	1	2	1	2	11778.2367	0.0028
6	1	6	5	0	5	11841.6862	-0.0132
4	2	3	3	1	2	12997.6067	0.0000
7	1	7	6	0	6	13367.3971	0.0004
8	0	8	7	1	7	13603.6214	0.0054
4	2	2	3	1	3	13890.8522	0.0086

Table S7: Observed transition frequencies (in MHz) for Ar-$^{13}CH_2CHCF_3$

J'	K_a'	K_c'	J''	K_a''	K_c''	Observed	Obs - Calc
8	1	8	7	0	7	14903.5697	0.0077
3	3	1	2	2	0	15540.7570	-0.0017
3	3	0	2	2	1	15547.5641	0.0076
6	2	5	5	1	4	16100.5020	0.0040
5	2	3	4	1	4	16130.5943	-0.0105
9	1	9	8	0	8	16461.9762	-0.0049
4	3	2	3	2	1	17376.6607	-0.0069
4	3	1	3	2	2	17410.9451	-0.0079

Table S8: Observed transition frequencies (in MHz) for Ar-$CH_2{}^{13}CHCF_3$

J'	K_a'	K_c'	J''	K_a''	K_c''	Observed	Obs - Calc
2	1	1	2	0	2	2243.6707	0.0071
5	1	4	5	0	5	3154.2090	0.0033
3	0	3	2	1	2	3666.3236	-0.0043
1	1	1	0	0	0	3835.8824	0.0072
7	2	5	7	1	6	5150.1879	-0.0015
5	2	3	5	1	4	5420.0979	0.0021
2	1	2	1	0	1	5560.4107	0.0019
4	2	2	4	1	3	5608.8077	-0.0049
4	0	4	3	1	3	5652.8822	0.0013
2	2	0	2	1	1	5959.6904	-0.0043
2	2	1	2	1	2	6333.6735	0.0027
3	2	2	3	1	3	6526.9136	-0.0034
4	2	3	4	1	4	6786.4365	0.0023
5	2	4	5	1	5	7113.3320	-0.0015
3	1	3	2	0	2	7223.7961	-0.0004
6	2	5	6	1	6	7508.4571	0.0066
5	0	5	4	1	4	7654.3899	0.0006
8	2	7	8	1	8	8503.5787	0.0003
4	1	4	3	0	3	8833.5875	0.0013
8	3	5	8	2	6	9525.2154	-0.0047
6	0	6	5	1	5	9652.6063	0.0077
2	2	1	1	1	0	9782.8393	-0.0016
2	2	0	1	1	1	9915.3239	-0.0014
6	3	3	6	2	4	9963.4942	0.0076
3	3	0	3	2	1	10214.1021	0.0009
4	3	2	4	2	3	10258.4333	-0.0007
5	3	3	5	2	4	10289.2125	-0.0005
5	1	5	4	0	4	10401.7285	0.0063
7	0	7	6	1	6	11631.0243	-0.0135
3	2	1	2	1	2	11916.1260	-0.0024
6	1	6	5	0	5	11943.6949	0.0109
4	2	3	3	1	2	13166.9142	-0.0020
4	2	2	3	1	3	14013.4865	-0.0073
3	3	1	2	2	0	15790.5213	-0.0028
3	3	0	2	2	1	15796.5599	0.0011
5	2	3	4	1	4	16228.6919	0.0010
6	2	5	5	1	4	16297.9217	-0.0071
9	1	9	8	0	8	16575.3463	0.0022
4	3	2	3	2	1	17628.6486	-0.0052
4	3	1	3	2	2	17659.0876	0.0087

Table S9: Observed transition frequencies (in MHz) for Ar-$CH_2CH^{13}CF_3$

J'	K_a'	K_c'	J''	K_a''	K_c''	Observed	Obs - Calc
4	1	3	4	0	4	2771.1188	-0.0020
5	1	4	5	0	5	3167.1799	-0.0018
3	0	3	2	1	2	3641.1459	0.0087
6	1	5	6	0	6	3678.8781	0.0148
1	1	1	0	0	0	3866.8989	-0.0038
7	2	5	7	1	6	5230.1281	0.0097
6	2	4	6	1	5	5343.7246	0.0021
5	2	3	5	1	4	5509.0183	-0.0006
2	1	2	1	0	1	5594.3854	0.0005
4	0	4	3	1	3	5629.0408	0.0033
4	2	2	4	1	3	5698.6600	-0.0026
3	2	2	3	1	2	5857.8458	-0.0034
3	2	1	3	1	2	5886.0502	-0.0032
2	2	0	2	1	1	6047.9224	0.0083
2	2	1	2	1	2	6417.9005	-0.0048
3	2	2	3	1	3	6608.9288	-0.0026
4	2	3	4	1	4	6865.4300	0.0001
5	2	4	5	1	5	7188.4876	-0.0019
3	1	3	2	0	2	7261.3375	0.0005
5	0	5	4	1	4	7632.7758	-0.0062
7	2	6	7	1	7	8037.1093	-0.0064
7	1	6	6	2	5	8355.6035	-0.0033
8	2	7	8	1	8	8562.5169	0.0009
4	1	4	3	0	3	8875.0347	0.0003
6	0	6	5	1	5	9634.5311	-0.0019
8	3	5	8	2	6	9688.3675	-0.0008
2	2	1	1	1	0	9872.9719	-0.0001
2	2	0	1	1	1	10003.8519	0.0013
6	3	3	6	2	4	10116.1781	-0.0036
5	3	2	5	2	3	10242.0347	0.0092
4	3	1	4	2	2	10318.6859	0.0004
3	3	1	3	2	2	10386.9089	-0.0059
5	1	5	4	0	4	10447.0003	-0.0043
6	3	4	6	2	5	10482.7427	0.0089
7	3	5	7	2	6	10563.0327	-0.0038
8	3	6	8	2	7	10679.6499	-0.0067
3	2	2	2	1	1	11600.1285	-0.0022
7	0	7	6	1	6	11617.9765	0.0011
6	1	6	5	0	5	11992.2398	0.0005
3	2	1	2	1	2	12003.9821	-0.0006
4	2	3	3	1	2	13263.6716	-0.0005
7	1	7	6	0	6	13527.4326	-0.0075
8	0	8	7	1	7	13570.9518	0.0045

Table S9: Observed transition frequencies (in MHz) for Ar-$CH_2CH^{13}CF_3$

J'	K_a'	K_c'	J''	K_a''	K_c''	Observed	Obs - Calc
4	2	2	3	1	3	14098.8211	0.0001
3	3	1	2	2	0	15939.1329	0.0017
3	3	0	2	2	1	15944.9534	0.0056
5	2	3	4	1	4	16308.9802	-0.0024
6	2	5	5	1	4	16404.0183	0.0125
4	3	2	3	2	1	17779.2482	-0.0058
4	3	1	3	2	2	17808.5747	-0.0005

Table S10: Observed transition frequencies (in MHz) for CH_2CHCF_3-HCCH

J'	K_a'	K_c'	J''	K_a''	K_c''	Observed	Obs - Calc
2	1	1	2	0	2	2116.1122	0.0028
2	0	2	1	1	1	2213.4482	0.0034
3	1	2	3	0	3	2414.7804	-0.0002
6	2	5	5	3	2	2555.3252	0.0004
4	1	3	4	0	4	2851.0537	0.0049
5	1	5	4	2	2	2886.7502	0.0021
8	3	6	7	4	3	3154.6823	0.0134
4	1	3	3	2	2	3261.6518	0.0042
5	1	4	5	0	5	3448.7785	0.0067
1	1	1	0	0	0	3751.5156	0.0077
6	1	6	5	2	3	4080.6431	0.0094
2	1	1	1	1	0	4157.0803	-0.0079
6	1	5	6	0	6	4223.7362	0.0052
3	0	3	2	1	2	4330.3234	0.0059
7	2	6	6	3	3	4373.0659	0.0085
6	2	4	6	1	5	4609.3719	0.0099
7	2	5	7	1	6	4642.8787	0.0086
5	2	3	5	1	4	4700.4275	0.0094
8	2	6	8	1	7	4834.1746	0.0090
4	2	2	4	1	3	4874.9028	0.0089
7	1	7	6	2	4	5026.4411	0.0097
3	2	1	3	1	2	5086.3876	0.0084
9	3	7	8	4	4	5157.0539	0.0126
7	1	6	7	0	7	5175.5127	0.0098
9	2	7	9	1	8	5209.2485	0.0117
2	2	0	2	1	1	5289.5453	0.0084
2	1	2	1	0	1	5571.3342	0.0094
9	3	6	8	4	5	5612.1832	-0.0026
11	4	8	10	5	5	5619.5801	0.0012
5	1	4	4	2	3	5635.8185	0.0092
8	1	8	7	2	5	5700.7318	-0.0030
3	1	3	2	1	2	5710.8896	-0.0012
7	2	5	6	3	4	5726.2084	0.0111
10	2	8	10	1	9	5787.0067	0.0167
2	2	1	2	1	2	5794.8615	0.0058
3	0	3	2	0	2	5929.0505	0.0099
3	2	2	2	2	1	5976.6895	0.0148
3	2	2	3	1	3	6060.6385	-0.0011
8	2	7	7	3	4	6086.3525	-0.0011
3	1	2	2	1	1	6227.7098	-0.0019
8	1	7	8	0	8	6284.2580	-0.0033
4	0	4	3	1	3	6471.2360	-0.0024
11	2	9	11	1	10	6576.1426	0.0013

Table S10: Observed transition frequencies (in MHz) for CH_2CHCF_3-HCCH

J'	K_a'	K_c'	J''	K_a''	K_c''	Observed	Obs - Calc
5	2	4	5	1	5	6869.9651	0.0004
12	3	9	12	2	10	6989.0706	-0.0121
11	3	8	11	2	9	7078.9930	-0.0060
13	3	10	13	2	11	7086.9235	-0.0134
10	3	8	9	4	5	7128.7413	-0.0036
3	1	3	2	0	2	7309.6123	-0.0018
10	3	7	10	2	8	7316.4925	0.0011
6	2	5	6	1	6	7415.4247	-0.0012
9	1	8	9	0	9	7514.0128	-0.0013
12	2	10	12	1	11	7570.1940	0.0048
4	1	4	3	1	3	7601.4971	-0.0037
9	3	6	9	2	7	7651.5345	-0.0003
4	0	4	3	0	3	7851.8111	-0.0007
10	3	7	9	4	6	7941.0407	-0.0018
15	3	12	15	2	13	7953.0533	-0.0084
4	2	3	3	2	2	7959.3451	-0.0097
8	3	5	8	2	6	8028.6611	0.0069
7	2	6	7	1	7	8053.4142	0.0022
6	1	5	5	2	4	8060.2345	-0.0018
8	2	6	7	3	5	8223.0272	-0.0017
4	1	3	3	1	2	8288.0759	-0.0040
7	3	4	7	2	5	8394.5635	0.0032
5	0	5	4	1	4	8605.5288	-0.0023
6	3	3	6	2	4	8706.7878	0.0018
13	2	11	13	1	12	8744.4643	0.0019
8	2	7	8	1	8	8780.1812	0.0020
10	1	9	10	0	10	8820.3157	0.0013
5	3	2	5	2	3	8940.7376	0.0026
4	1	4	3	0	3	8982.0716	-0.0026
10	2	9	9	3	6	9043.3029	0.0010
11	3	9	10	4	6	9048.1043	0.0041
4	3	1	4	2	2	9092.4137	0.0000
3	3	0	3	2	1	9174.8898	-0.0027
3	3	1	3	2	2	9234.3068	-0.0033
4	3	2	4	2	3	9265.9052	-0.0015
5	3	3	5	2	4	9328.3657	-0.0029
6	3	4	6	2	5	9434.3376	0.0031
2	2	1	1	1	0	9434.5556	-0.0021
5	1	5	4	1	4	9482.4249	-0.0035
9	2	8	9	1	9	9589.6045	0.0031
7	3	5	7	2	6	9596.5707	0.0023
2	2	0	1	1	1	9619.0896	-0.0016
5	0	5	4	0	4	9735.7908	-0.0026

Table S10: Observed transition frequencies (in MHz) for CH_2CHCF_3-HCCH

J'	K_a'	K_c'	J''	K_a''	K_c''	Observed	Obs - Calc
8	3	6	8	2	7	9827.0182	0.0062
5	2	4	4	2	3	9933.8974	-0.0021
5	3	3	4	3	2	9996.3563	-0.0052
5	3	2	4	3	1	10007.3552	-0.0068
14	2	12	14	1	13	10058.1741	0.0087
9	3	7	9	2	8	10135.9568	0.0059
5	2	3	4	2	2	10159.0412	0.0005
11	1	10	11	0	11	10159.6156	-0.0088
5	1	4	4	1	3	10333.5246	0.0081
11	3	8	10	4	7	10395.4071	-0.0085
10	2	9	10	1	10	10473.4316	-0.0037
7	1	6	6	2	5	10517.6649	0.0031
5	1	5	4	0	4	10612.6886	-0.0022
6	0	6	5	1	5	10706.4574	-0.0126
9	2	7	8	3	6	10818.4644	-0.0025
11	3	9	11	2	10	11018.3059	-0.0030
6	1	6	5	1	5	11352.9131	-0.0132
11	4	7	11	3	8	11801.0179	-0.0053
3	2	1	2	1	2	11831.4773	0.0000
6	2	5	5	2	4	11898.3980	0.0103
10	4	6	10	3	7	12170.2909	0.0075
6	1	6	5	0	5	12229.8226	-0.0011
6	2	4	5	2	3	12267.2679	-0.0026
6	1	5	5	1	4	12358.3228	-0.0037
12	2	11	12	1	12	12424.4308	-0.0075
9	4	5	9	3	6	12445.9076	0.0109
8	4	4	8	3	5	12636.3212	0.0028
7	0	7	6	1	6	12757.5837	-0.0011
7	4	3	7	3	4	12758.7008	0.0042
6	4	2	6	3	3	12832.0292	0.0035
8	4	5	8	3	6	12857.8222	0.0062
7	4	4	7	3	5	12863.0302	0.0048
9	4	6	9	3	7	12867.6554	0.0073
5	4	1	5	3	2	12872.8914	-0.0009
6	4	3	6	3	4	12874.8256	-0.0012
5	4	2	5	3	3	12887.4126	-0.0012
4	4	0	4	3	1	12893.8724	0.0039
4	4	1	4	3	2	12897.5288	-0.0142
10	4	7	10	3	8	12903.2980	-0.0009
11	4	8	11	3	9	12977.1933	-0.0082
8	1	7	7	2	6	12985.4296	-0.0020
4	2	3	3	1	2	12985.7849	-0.0022
7	1	7	6	0	6	13859.5248	0.0003

Table S10: Observed transition frequencies (in MHz) for CH_2CHCF_3-HCCH

J'	K_a'	K_c'	J''	K_a''	K_c''	Observed	Obs - Calc
4	2	2	3	1	3	14197.1778	-0.0032
5	2	4	4	1	3	14631.6049	-0.0018
8	0	8	7	1	7	14754.5828	0.0005
3	3	1	2	2	0	15198.9134	-0.0040
3	3	0	2	2	1	15211.5143	0.0003
9	1	8	8	2	7	15436.2434	0.0013
8	1	8	7	0	7	15519.1066	-0.0010
6	2	5	5	1	4	16196.4767	-0.0011
9	5	4	9	4	5	16474.9808	0.0034
9	5	5	9	4	6	16498.3942	-0.0018
8	5	3	8	4	4	16521.4434	0.0101
7	5	2	7	4	3	16552.1385	-0.0020
7	5	3	7	4	4	16555.2321	-0.0078
6	5	1	6	4	2	16572.0067	0.0125
6	5	2	6	4	3	16572.8381	-0.0102
9	0	9	8	1	8	16702.3994	-0.0077
5	2	3	4	1	4	16754.7182	-0.0027
4	3	2	3	2	1	17165.3105	-0.0041
9	1	9	8	0	8	17214.8939	0.0054
4	3	1	3	2	2	17228.9416	-0.0135
7	2	6	6	1	5	17689.2113	0.0059
10	1	9	9	2	8	17841.1553	-0.0048

Table S11: Observed transition frequencies (in MHz) for $^{13}CH_2CHCF_3$-HCCH

J'	K_a'	K_c'	J''	K_a''	K_c''	Observed	Obs - Calc
4	0	4	3	1	3	6477.9952	0.0001
3	1	3	2	0	2	7186.0774	-0.0025
5	0	5	4	1	4	8590.2941	0.0014
4	1	4	3	0	3	8834.9129	-0.0001
2	2	1	1	1	0	9213.7697	-0.0022
2	2	0	1	1	1	9405.7925	0.0003
5	1	5	4	0	4	10443.5479	-0.0006
6	0	6	5	1	5	10663.4021	-0.0009
3	2	2	2	1	1	11013.0525	0.0035
6	1	6	5	0	5	12042.4735	-0.0014
7	0	7	6	1	6	12682.4643	0.0013
4	2	3	3	1	2	12721.1584	-0.0012
7	1	7	6	0	6	13658.3803	0.0010
5	2	4	4	1	3	14340.5568	0.0008
8	0	8	7	1	7	14645.7594	-0.0005
3	3	1	2	2	0	14840.2433	0.0018
3	3	0	2	2	1	14854.2719	0.0007
8	1	8	7	0	7	15307.3814	0.0010
6	2	5	5	1	4	15876.8974	0.0009
9	0	9	8	1	8	16560.4814	-0.0004
4	3	2	3	2	1	16789.6607	0.0013
4	3	1	3	2	2	16860.5533	-0.0041
9	1	9	8	0	8	16993.6849	-0.0005

Table S12: Observed transition frequencies (in MHz) for $CH_2{}^{13}CHCF_3$-HCCH

J'	K_a'	K_c'	J''	K_a''	K_c''	Observed	Obs - Calc
4	0	4	3	1	3	6479.8400	0.0002
5	0	5	4	1	4	8609.0170	0.0007
4	1	4	3	0	3	8942.2143	-0.0018
2	2	1	1	1	0	9369.4920	0.0020
2	2	0	1	1	1	9556.4753	-0.0024
5	1	5	4	0	4	10567.3030	-0.0011
6	0	6	5	1	5	10702.9665	0.0003
3	2	2	2	1	1	11184.2258	-0.0003
6	1	6	5	0	5	12180.0725	0.0005
7	0	7	6	1	6	12745.6763	0.0003
4	2	3	3	1	2	12909.9550	0.0008
7	1	7	6	0	6	13806.8176	-0.0009
5	2	4	4	1	3	14548.8972	0.0009
8	0	8	7	1	7	14733.6502	0.0015
3	3	1	2	2	0	15092.9342	-0.0002
3	3	0	2	2	1	15105.9824	0.0030
8	1	8	7	0	7	15464.5259	-0.0004
6	2	5	5	1	4	16106.1683	0.0009
9	0	9	8	1	8	16672.5534	-0.0022
4	3	2	3	2	1	17055.6318	-0.0013
4	3	1	3	2	2	17121.5421	-0.0019
9	1	9	8	0	8	17158.8624	0.0014

Table S13: Observed transition frequencies (in MHz) for $CH_2CH^{13}CF_3$-HCCH

J'	K_a'	K_c'	J''	K_a''	K_c''	Observed	Obs - Calc
4	0	4	3	1	3	6459.6408	-0.0008
3	1	3	2	0	2	7302.5532	-0.0025
5	0	5	4	1	4	8591.2667	0.0007
4	1	4	3	0	3	8972.8064	0.0025
2	2	1	1	1	0	9429.9966	0.0017
2	2	0	1	1	1	9614.1631	0.0007
5	1	5	4	0	4	10601.2019	-0.0003
6	0	6	5	1	5	10689.7301	-0.0004
3	2	2	2	1	1	11247.1483	-0.0005
6	1	6	5	0	5	12216.0265	0.0006
7	0	7	6	1	6	12738.5395	0.0000
4	2	3	3	1	2	12976.5227	-0.0024
7	1	7	6	0	6	13843.2823	0.0001
5	2	4	4	1	3	14620.2332	0.0000
8	0	8	7	1	7	14733.3337	0.0004
3	3	1	2	2	0	15191.9805	-0.0006
3	3	0	2	2	1	15204.5275	-0.0009
8	1	8	7	0	7	15500.2999	0.0004
6	2	5	5	1	4	16183.1236	0.0002
9	0	9	8	1	8	16678.9878	-0.0002
4	3	2	3	2	1	17155.7258	-0.0006
9	1	9	8	0	8	17193.4443	-0.0006
4	3	1	3	2	2	17219.1191	0.0021

Table S14: Observed transition frequencies (in MHz) for CH_2CHCF_3-$H^{13}CCH$

J'	K_a'	K_c'	J''	K_a''	K_c''	Observed	Obs - Calc
4	0	4	3	1	3	6315.0822	-0.0001
3	1	3	2	0	2	7237.9629	0.0011
5	0	5	4	1	4	8417.4134	-0.0008
4	1	4	3	0	3	8885.5822	-0.0005
2	2	1	1	1	0	9412.4963	-0.0022
2	2	0	1	1	1	9590.6057	0.0016
6	0	6	5	1	5	10490.1055	-0.0008
5	1	5	4	0	4	10491.0789	0.0004
3	2	2	2	1	1	11203.4844	-0.0014
3	2	1	2	1	2	11759.8833	0.0005
6	1	6	5	0	5	12081.2487	0.0007
7	0	7	6	1	6	12516.1716	0.0010
4	2	3	3	1	2	12909.4222	0.0016
7	1	7	6	0	6	13681.3387	-0.0010
8	0	8	7	1	7	14490.0021	-0.0001
5	2	4	4	1	3	14532.2283	-0.0001
3	3	1	2	2	0	15169.2430	0.0004
3	3	0	2	2	1	15180.9270	-0.0009
8	1	8	7	0	7	15308.9704	0.0010
6	2	5	5	1	4	16076.3257	-0.0009
9	0	9	8	1	8	16415.1818	0.0001
9	1	9	8	0	8	16971.4912	-0.0005
4	3	2	3	2	1	17103.2883	0.0014
4	3	1	3	2	2	17162.3135	-0.0007

Table S15: Observed transition frequencies (in MHz) for CH_2CHCF_3-$HC^{13}CH$

J'	K_a'	K_c'	J''	K_a''	K_c''	Observed	Obs - Calc
4	0	4	3	1	3	6234.6288	0.0009
3	1	3	2	0	2	7199.2451	-0.0009
5	0	5	4	1	4	8320.0458	0.0017
2	2	1	1	1	0	9398.3240	0.0044
2	2	0	1	1	1	9573.3159	-0.0004
6	0	6	5	1	5	10377.5933	-0.0007
5	1	5	4	0	4	10425.8481	0.0009
3	2	2	2	1	1	11174.2338	-0.0034
6	1	6	5	0	5	12001.8725	-0.0008
7	0	7	6	1	6	12390.0972	-0.0017
4	2	3	3	1	2	12866.5054	-0.0019
7	1	7	6	0	6	13586.5576	0.0004
8	0	8	7	1	7	14351.3602	0.0005
5	2	4	4	1	3	14476.9671	0.0010
3	3	1	2	2	0	15149.4541	0.0006
3	3	0	2	2	1	15160.7138	0.0002
8	1	8	7	0	7	15197.6074	-0.0007
6	2	5	5	1	4	16009.8235	0.0013
9	0	9	8	1	8	16264.3006	0.0004
9	1	9	8	0	8	16842.8488	0.0001
4	3	2	3	2	1	17066.5801	0.0002
4	3	1	3	2	2	17123.4531	-0.0011

Table S16. Interaction lengths in Ångstroms between Ar and heavy atoms in three isomers of Ar-TFP obtained from *ab initio* calculations. The experimental results come from a fit to the moments of inertia of four isotopologues of Ar-TFP.

	No BSSE Correction	BSSE Correction	No BSSE Correction	BSSE Correction	No BSSE Correction	BSSE Correction	Experiment
	Isomer (a)		Isomer (b)		Isomer (c)		
Ar−C1	3.880	3.976	5.218	5.350	6.178	6.412	3.8932(16)
Ar−C2	4.001	4.151	3.949	4.095	5.335	5.574	4.1202(50)
Ar−C3	3.863	4.072	3.894	4.087	3.846	3.9112	3.91156(30)
Ar−F1	3.512	3.726	5.201	5.399	3.561	3.783	3.3525(93)
Ar−F2	3.526	3.746	3.556	3.752	3.569	3.794	3.7267(78)
Ar−F3	5.172	5.389	3.556	3.752	3.569	3.794	5.1956(29)

Table S17. Rotational constants, dipole moment components, and relative equilibrium and zero-point corrected energies for three isomers of Ar-TFP obtained from *ab initio* calculations at the MP2/6-311++G(2d,2p) level without and with BSSE correction.

	No BSSE Correction	BSSE Correction	No BSSE Correction	BSSE Correction	No BSSE Correction	BSSE Correction
	Isomer (a)		Isomer (b)		Isomer (c)	
A/MHz	2986	3013	3153	3122	4913	4898
B/MHz	1010	926	904	841	798	733
C/MHz	881	818	801	749	780	718
$\|\mu_a\|$ / D	0.415	0.509	0.319	0.231	2.578	2.569
$\|\mu_b\|$ / D	2.380	2.367	2.453	2.472	0.126	0.149
$\|\mu_c\|$ / D	0.641	0.643	0.000	0.000	0.000	0.000
$E_{equil}^{a,b}$ / cm^{-1}	0.0	0.0	18.6	14.2	83.2	58.3
$E_{zpe}^{a,c}$ / cm^{-1}	0.0	0.0	26.6	18.8	83.8	61.1

[a]The energies for each calculation method are given relative to the values obtained using the same calculation method for the most stable isomer. These are −941.881376 Hartree, −941.880670 Hartree, −941.823724 Hartree, and −941.822940 Hartree for E_{equil}, E_{equil}+BSSE, E_{zpe}, and E_{zpe}+BSSE, respectively.

[b]This equilibrium energy is determined by using the average structure of TFP[20] and optimizing the intermolecular degrees of freedom with argon, without and with BSSE correction, respectively.

[c]The equilibrium structure and energy of the complex are calculated while allowing a full relaxation of the complex geometry, including the structural parameters of TFP. A harmonic zero-point correction to the energy is calculated for this structure, which is included in both columns for each isomer, as is a counterpoise correction for BSSE, which is included in the second column for each.

Table S18. Rotational constants, dipole moment components, and relative equilibrium and zero-point corrected energies for three isomers of HCCH-TFP obtained from *ab initio* calculations at the MP2/6-311++G(2d,2p) level without and with BSSE correction.

	No BSSE Correction	BSSE Correction	No BSSE Correction	BSSE Correction	No BSSE Correction	BSSE Correction
	Isomer (i)		Isomer (ii)		Isomer (iii)	
A/MHz	2842	2821	3473	3478	3039	2989
B/MHz	1132	1058	806	780	1073	1022
C/MHz	944	895	772	748	937	894
$\|\mu_a\|$ / D	0.296	0.441	2.540	2.532	0.649	0.584
$\|\mu_b\|$ / D	2.454	2.447	1.013	1.016	2.377	2.387
$\|\mu_c\|$ / D	0.000	0.019	0.000	0.000	0.608	0.648
$E_{\text{equil}}^{a,b}$ / cm^{-1}	0.0	0.0	129.4	33.1	136.8	145.2
$E_{\text{zpe}}^{a,c}$ / cm^{-1}	0.0	0.0	131.2	15.3	105.8	106.9

[a]The energies for each calculation method are given relative to the values obtained using the same calculation method for the most stable isomer. These are −492.038106 Hartree, −492.036749 Hartree, −491.953788 Hartree, and −491.952281 Hartree for E_{equil}, E_{equil}+BSSE, E_{zpe}, and E_{zpe}+BSSE, respectively.

[b]This equilibrium energy is determined by using the average structures of TFP[20] and HCCH[21] and optimizing the intermolecular degrees of freedom between TFP and HCCH, without and with BSSE correction, respectively.

[c]The equilibrium structure and energy of the complex are calculated while allowing a full relaxation of the complex geometry, including the structural parameters of TFP and HCCH. A harmonic zero-point correction to the energy is calculated for this structure, which is included in both columns for each isomer, as is a counterpoise correction for BSSE, which is included in the second column for each.

Table S19. Spectroscopic constants (in MHz, unless otherwise noted) for four isotopologues of TFP determined using the Watson *S* reduced Hamiltonian in the I^r representation.[a]

	CH_2CHCF_3	$^{13}CH_2CHCF_3$	$CH_2{}^{13}CHCF_3$	$CH_2CH^{13}CF_3$
A	5442.70336(66)	5442.8309(13)	5415.8502(13)	5443.2309(12)
B	2943.47602(45)	2863.45324(99)	2924.46303(96)	2942.33307(91)
C	2873.16044(45)	2796.88815(98)	2847.67956(95)	2872.05950(90)
D_J / 10^{-3}	0.418(14)	0.280(62)	0.450(60)	0.481(56)
D_{JK} / 10^{-3}	6.3512(31)	6.409(23)	5.961(29)	6.334(21)
D_K / 10^{-3}	−5.388(22)	−5.074(97)	−4.717(93)	−5.326(77)
d_1 [b] / 10^{-3}	0.007737(95)	[0.007737]	[0.007737]	[0.007737]
d_2 / 10^{-3}	0.023768(46)	0.02483(88)	0.0242(14)	0.02471(94)
No. of rot. transitions	98	35	34	37
No. of *a* type	14	9	9	9
No. of *b* type	84	26	25	28
J range	0 – 16	0 – 8	0 – 7	0 – 8
K_a range	0 – 4	0 – 3	0 – 3	0 – 3
rms/kHz	5.71	5.27	5.09	4.83

[a] 1σ standard deviations in the parameters are given in parentheses.

[b] The value of d_1 for each of the isotopologue singly substituted with ^{13}C is fixed to that for the most abundant isotopologue and enclosed by square brackets.

Table S20. Spectroscopic constants (in MHz, unless otherwise noted) for four isotopologues of TFP determined using the Watson *A* reduced Hamiltonian in the I^r representation.[a]

	CH_2CHCF_3	$^{13}CH_2CHCF_3$	$CH_2{}^{13}CHCF_3$	$CH_2CH^{13}CF_3$
A	5442.70356(66)	5442.8311(13)	5415.8504(13)	5443.2311(12)
B	2943.46223(45)	2863.4376(11)	2924.4502(12)	2942.3187(10)
C	2873.17405(45)	2796.9036(11)	2847.6923(12)	2872.0737(10)
Δ_J / 10^{-3}	0.371(14)	0.231(62)	0.401(60)	0.432(56)
Δ_{JK} / 10^{-3}	6.6325(32)	6.705(28)	6.251(37)	6.630(28)
Δ_K / 10^{-3}	−5.617(22)	−5.318(98)	−4.958(99)	−5.571(80)
δ_J [b] / 10^{-3}	−0.007763(96)	[−0.007763]	[−0.007763]	[−0.007763]
δ_K / 10^{-3}	−6.850(13)	−7.79(28)	−6.39(37)	−7.14(27)
No. of rot. transitions	98	35	34	37
No. of *a* type	14	9	9	9
No. of *b* type	84	26	25	28
J range	0 – 16	0 – 8	0 – 7	0 – 8
K_a range	0 – 4	0 – 3	0 – 3	0 – 3
rms/kHz	5.71	5.27	5.09	4.83

[a] 1σ standard deviations in the parameters are given in parentheses.

[b] The value of δ_J for each of the isotopologue singly substituted with ^{13}C is fixed to that for the most abundant isotopologue and enclosed by square brackets.

Table S21. Spectroscopic constants (in MHz, unless otherwise noted) for the most abundant isotopologue of Ar-TFP with and without the inclusion of the sextic centrifugal distortion constant h_1.[a]

	with h_1	without h_1
A	3003.64088(17)	3003.64072(23)
B	991.865340(69)	991.865793(88)
C	866.022410(68)	866.021950(86)
D_J / 10^{-3}	2.48278(59)	2.48251(82)
D_{JK} / 10^{-3}	13.0958(29)	13.0904(40)
D_K / 10^{-3}	–9.7445(68)	–9.7423(93)
d_1 / 10^{-3}	–0.37245(20)	–0.375790(60)
d_2 / 10^{-3}	–0.040501(15)	–0.040589(20)
H_J / 10^{-6}	–0.0494(16)	0.0510(22)
H_{JK} / 10^{-6}	–0.443(12)	-0.474(17)
h_1 / 10^{-6}	–0.01017(60)	--
No. of rot. transitions	320	320
No. of *a* type	59	59
No. of *b* type	177	177
No. of *c* type	84	84
J range	0 – 18	0 – 18
K_a range	0 – 5	0 – 5
rms/kHz	3.66	5.04

[a]1σ standard deviations in the parameters are given in parentheses.

Table S22: Principal coordinates for the experimental structures of the argon and acetylene complexes of 3,3,3-trifluoropropene

Ar-3,3,3-trifluoropropene

	a	*da*	*b*	*db*	*c*	*dc*
C	-0.44150	0.00194	2.15877	0.00045	-0.02529	0.00009
C	-1.16580	0.00528	1.19994	0.00152	0.54474	0.00372
C	-1.22308	0.00027	-0.19498	0.00026	0.02414	0.00021
F	-0.47556	0.00958	-0.38764	0.00298	-1.07116	0.00696
F	-0.79937	0.00814	-1.07959	0.00277	0.94737	0.00608
F	-2.48345	0.00281	-0.54648	0.00189	-0.29576	0.01034
H	0.14584	0.00765	1.95490	0.00251	-0.90840	0.00571
H	-0.42946	0.00516	3.15709	0.00133	0.38653	0.00285
H	-1.76227	0.01310	1.37703	0.00395	1.42873	0.00951
Ar	2.68827	0.00014	-0.15633	0.00043	0.01336	0.00009

HCCH-3,3,3-trifluoropropene

	a	*da*	*b*	*db*	*c*	*dc*
C	-1.38201	0.01228	-2.12312	0.00390	0.00000	0.00000
C	-0.46432	0.00676	-1.16045	0.00137	0.00000	0.00000
C	-0.80634	0.00156	0.28977	0.00060	0.00000	0.00000
F	-2.12504	0.00293	0.52774	0.00816	0.00000	0.00000
F	-0.29652	0.00514	0.91273	0.00233	-1.08001	0.00000
F	-0.29652	0.00514	0.91273	0.00233	1.08001	0.00000
H	-2.43463	0.01090	-1.88146	0.00994	0.00000	0.00000
H	-1.08849	0.01825	-3.16247	0.00222	0.00000	0.00000
H	0.59524	0.00799	-1.37467	0.00745	0.00000	0.00000
H	2.32985	0.01601	1.24417	0.00788	0.00000	0.00000
C	2.96124	0.00575	0.39577	0.00064	0.00000	0.00000
C	3.68262	0.00599	-0.57355	0.00853	0.00000	0.00000
H	4.31400	0.01626	-1.42196	0.01616	0.00000	0.00000
